# An account of *Colletotrichum* species associated with anthracnose of *Atractylodes ovata* in South Korea based on morphology and molecular data

**DOI:** 10.1371/journal.pone.0263084

**Published:** 2022-01-25

**Authors:** Oliul Hassan, Ju Sung Kim, Bekale Be Ndong Dimitri Romain, Taehyun Chang

**Affiliations:** Department of Ecology & Environmental System, College of Ecology & Environmental Sciences, Kyungpook National University, Sangju, Gyeongbuk, Republic of Korea; Bangabandhu Sheikh Mujibur Rahman Agricultural University, BANGLADESH

## Abstract

Ovate-leaf atractylodes (OLA) (*Atractylodes ovata*) is a well-known medicinal plant in Korea; its dried rhizome and root extracts are used in herbal medicine. However, anthracnose is a great challenge to the OLA cultivation in South Korea. *Colletotrichum* spp. is a major group of plant pathogens responsible for anthracnose on a range of economically important hosts. Its occurrence on OLA remains unresolved. To investigate the diversity, morphology, phylogeny, and biology of *Colletotrichum* spp., 32 fungal isolates were obtained from 30 OLA-affected leaves collected from five different farms, in two regions in South Korea, Mungyeong and Sangju. The phylogenetic analysis with four or five gene loci (*ITS*, *TUB2*, *ACT*, *GAPDH*, and *CHS-1*) along with morphology of 26 representative isolates delineated six previously known *Colletotrichum* species including *C*. *fructicola*, *C*. *gloeosporioides* sensu stricto (*s*.s), *C*. *cigarro*, *C*. *plurivorum*, *C*. *siamense* and *C*. *sojae*, and one new species, described here as *C*. *ovataense*. Amongst these species, *C*. *gloeosporioides* s.s. and *C*. *plurivorum* were the most prevalent species. A pathogenicity test on the detached leaves revealed that different *Colletotrichum* species presented a distinct degree of virulence, confirming Koch’s postulates. In this study, *C*. *fructicola*, *C*. *cigarro*, *C*. *plurivorum*, *C*. *siamense*, and *C*. *sojae* were reported from *A*. *ovata* for the first time, as the causal agent of ovate-leaf atractylodes anthracnose. Understanding the diversity and biology of the *Colletotrichum* species population will help in managing this disease.

## Introduction

Ovate-leaf atractylodes (OLA) (*Atractylodes ovata* De Candolle) is one of the major cultivated species of genus atractylodes in South Korea [1]. This species is well known for its medicinal value. The rhizome extract of OLA is used for treating gastroduodenal diseases, inflammation, and cancer [1–3]. In recent years, the cultivation of this crop became more challenging due to the increased incidence of anthracnose. In 2019, approximately 60% of OLA plants in Mungyong and Sangju, South Korea, were affected by this disease. The identification of its causal agent is crucial to develop sustainable management strategies to control disease. A few *Colletotrichum* spp. has been reported as the causal agent of OLA anthracnose worldwide. The *Colletotrichum gloeosporioides* senso strico (s.s) is the only causal agent of anthracnose of *A*. *ovata* and *A*. *japonica* in South Korea [4]. *Colletotrichum spaethianum* was isolated from *A*. *japonica* in China [5]. However, the *Colletotrichum* spp. associated with OLA anthracnose remained largely unresolved.

Leaf spot or antharchnose is a destructive disease of many crops and one of the most encountered problems for farmers [6]. This disease reduces the plant vigor and yield by interrupting photosynthesis [7, 8]. Sunflower affected by leaf spot disease produces between16% to 65% less seeds per head with a lower seed weight (15% to 79%) [9]. Between 32% and 57% yield loss was recorded in mustard affected by this disease [10]). The affected plant also became more susceptible to other diseases and pests [7].

Different species of the genus *Colletotrichum* Corda are the most important plant pathogenic fungi causing anthracnose in numerous plant host worldwide [6, 11–13]. In recent year, *Colletotrichum* spp. is reported as leaf spot or anthrachnose pathogens in many crops such as *Ixora coccinea*, *Polygonatum odoratum*, *Liriope cymbidiomorpha*, *Hibiscus*, *Geranium*, and *Primula* hybrids [6, 11–13]. In Korea, *Colletotrichum* species were isolated from many host plants including persimmon, apple and peach [14–16].

According to recent results, 14 *Colletotrichum* species complexes and 15 singleton species have been identified in this genus [17, 18]. More than one *Colletotrichum* species has been reported as the causal agent of anthracnose is the same plant species including apple, peach, plum, and pear [15–17, 19, 20]. The old identification method of fungal species based on morphological characteristics is no longer considered to be effective [17, 18]. Advanced approaches, including the analysis of morphological and molecular characteristics, are currently the best choice and highly regarded by mycologists to identify *Colletotrichum* species.

So, Morphological and molecular characterization of the *Colletotrichum* spp. associated with OLA will provide a better understanding of the biology of this important genus. Aiming for the development of effective management strategies against anthracnose, the objectives of this study are: (i) to identify the diversity of *Colletotrichum* species associated with anthracnose of ovate-leaf atractylodes in South Korea by analyzing morphological characteristic coupled with a multigene phylogenetic approach, and (ii) to evaluate the pathogenicity of the identified *Colletotrichum* species.

## Materials and methods

### Sampling and isolation

**Fig 1:**
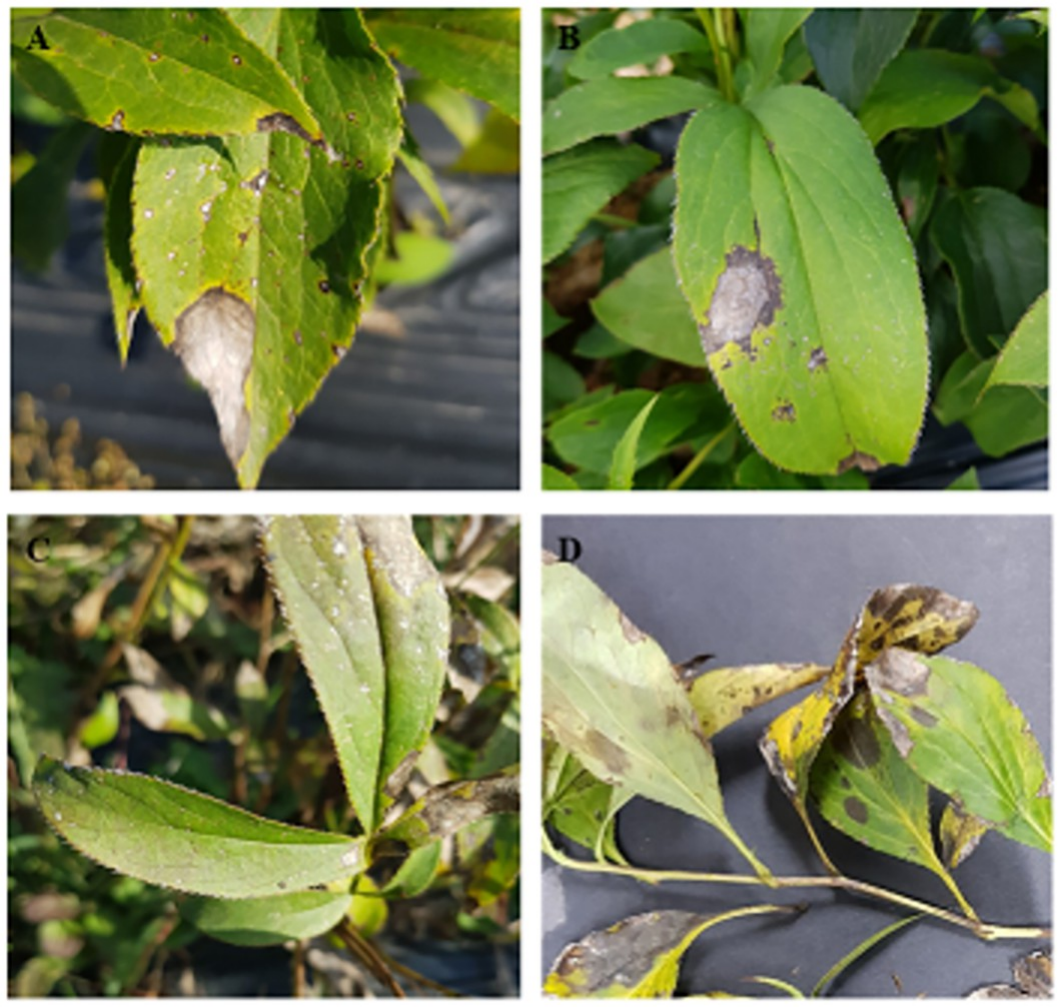
Symptoms of OLA anthracnose on leaves in the field. A-C: Symptoms of big necrotic lesion and small spots. D: Coalesce of small and big spots.

In August and September in 2019 and 2020, a survey was conducted in two farmer fields (August and September in 2019 and 2020) in Mungyeong and Sangju, South Korea. Symptoms observed on leaves were of two types, namely, big necrotic lesions and small spots. Big necrotic lesions had an irregular shape, greyish-white in the center with black margins and were > 13 mm covering 80% of the leaf blade (Fig 1A, 1B and 1D). Small spots were also greyish-white color with black margins and also had an irregular in shape, but were only 1.2–7 mm diameter, (Fig 1C). Acervuli or conidial masses were not found on any of the symptoms. The stems and inflorescences did not show any typical symptoms of anthracnose. The disease incidence was estimated as the proportion of leaves having at least one spot (as described above) per plant. To estimate disease incidence, 100 plants per field were selected. The disease incidence in both fields was of 45%–60%.

Leaves showing the aforementioned symptoms were collected from the surveyed field. Fungi were isolated from leaves presenting both symptom types. The tissues (5 mm^2^) containing the leaf lesion and neighboring asymptomatic regions were cut from the diseased leaves, its surface was sterilized with 1% NaOCl for one minute and 70% ethanol for 30s, and then rinsed with sterile distilled water before air dried. Disinfected excised tissue pieces were placed on potato dextrose agar (PDA, Difco Becton Dickinson) supplemented with tetracycline (0.05 g/L) and incubated at 25ºC in the dark. Newly emerging hyphal tips were transferred to a fresh PDA. This process (hyphal tipping) was repeated twice. Pure cultures were preserved in 15% glycerol at –80°C, for further use. Ex-type living culture of the representative isolates were deposited at Korean Agricultural Culture Collection (KACC). No permits were required to access as the farmer fields were rented during the study period.

### DNA extraction, PCR amplification and sequencing

**Fig 2:**
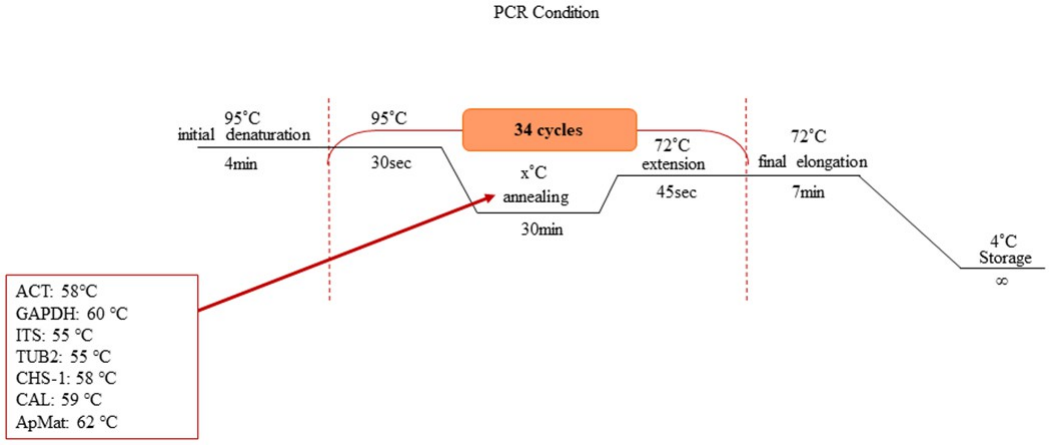
PCR reaction conditions. Annealing temperature of corresponding gene is given in the box.

Seven-days-old cultures were subject to genomic DNA extraction using the HiGene Genomic DNA Prep Kit (BIOFACT, Yuseong-Gu, and Daejeon, Korea) and following the manufacturer’s instruction. Seven loci, including the internal transcribed spacers (ITS), glyceraldehyde-3-phosphate dehydrogenase (*GAPDH*), partial actin (*ACT*), beta-tubulin (*TUB2*), chitin synthase (*CHS-1*), and Apn2–Mat1–2 intergenic spacer and the partial mating type (Mat1–2) gene (ApMat), were amplified by primer sets ITS1/ITS4 [21], GDF/GDR [22], ACT-512F/ACT-783R [23], Bt2a/Bt2b [24], CHS-79F/CHS-345R [23], and AM-F/AM-R [25], respectively. The conditions for PCR reaction were adopted from Hassan et al. [20] with some modification in annealing temperature. Details of the PCR condition shown in Fig 2.

The resulting PCR amplicons were purified and sequenced commercially using Macrogen, Inc. (Seoul, Korea). A consensus sequence was obtained by assembling forward and reverse sequences of each gene of each isolate with SeqMan version 7.1, Lasergene package (DNASTAR, Inc. Madison, WI). The sequences were deposited in GenBank (Table 1).

**Table 1:** List of 32 representative isolates of seven *Colletotrichum* spp. collected from leaf spot of OLA in South Korea, with collection details and GenBank accession numbers.

Species	Isolates No.	Location	GenBank accession number					
			*ITS*	*GAPDH*	*ACT*	*CHS*	*TUB*	*ApMat*
*C*. *fructicola*	B41	Sangju	LC604476	LC604540	LC604566	LC604592	LC604508	--
	3C2	Sangju	LC604477	LC604541	LC604567	LC604593	LC604509	--
	M742	Mungyeong	LC604478	LC604542	LC604568	LC604594	LC604510	--
	WHT1112	Mungyeong	LC604479	--	--	--	LC604511	--
	WHT181	Mungyeong	LC604480	LC604543	LC604569	LC604595	LC604512	--
	M52	Mungyeong	LC604481	--	--	--	LC604513	--
*C*. *gloeosporioides s*.s.	T74	Sangju	LC604482	LC604544	LC604570	LC604596	LC604514	--
	B1B3	Sangju	LC604483	LC604545	LC604571	LC604597	LC604515	--
	SPL912	Sangju	LC604484	LC604546	LC604572	LC604598	LC604516	--
	B2A3	Sangju	LC604485	LC604547	LC604573	LC604599	LC604517	--
	M123	Mungyeong	LC604486	LC604549	LC604575	LC604601	LC604518	--
	U1232	Mungyeong	LC604487	LC604548	LC604574	LC604600	LC604519	--
	WSH43	Mungyeong	LC604488	--	--	--	LC604520	--
*C*. *cigarro*	M92	Mungyeong	LC604489	LC604550	LC604576	LC604602	LC604521	LC637576
	SPL93	Sanju	LC604490	LC604551	LC604577	LC604603	LC604522	LC637577
*C*. *ovataense*	T71	Mungyeong	LC604491	LC604552	LC604578	LC604604	LC604523	--
	T72	Mungyeong	LC604492	LC604553	LC604579	LC604605	LC604524	--
	T77	Mungyeong	LC604493	LC604554	LC604580	LC604606	LC604525	--
*C*. *siamense*	M63	Mungyeong	LC604494	--	--	--	LC604526	--
	SPL213	Sangju	LC604495	LC604555	LC604581	LC604607	LC604527	LC604618
	SPL2133	Sangju	LC604496	LC604556	LC604582	LC604608	LC604528	LC604619
	SPL2136	Sangju	LC604497	LC604557	LC604583	LC604609	LC604529	LC604620
	SPL2139	Sangju	LC604498	LC604558	LC604584	LC604610	LC604530	LC604621
*C*. *sojae*	T2B2	Mungyong	LC604499	--	--	--	LC604531	--
	M72	Mungyong	LC604500	LC604559	LC604585	LC604611	LC604532	--
	SPL224	Sangju	LC604502	LC604561	LC604587	LC604613	LC604534	--
	SPL251	Sangju	LC604501	LC604560	LC604586	LC604612	LC604536	--
*C*. *plurivorum*	SPML22	Mungyeong	LC604503	LC604562	LC604588	LC604614	LC604535	--
	M34	Mungyeong	LC604504	LC604563	LC604589	LC604615	LC604536	--
	M45	Mungyeong	LC604506	LC604564	LC604590	LC604616	LC604538	--
	M54	Mungyeong	LC604507	LC604565	LC604591	LC604617	LC604539	--
	M412	Mungyeong	LC604505	--	--		LC604537	--

### Phylogenetic analyses

In order to identify the present isolates at species level sequences of amplified locus of reference isolates downloaded from GenBank (S1 and S2 Tables). Multiple sequences of each locus were aligned using MEGA v.6 [26] with the muscle multiple sequence alignment program set as default. The aligned sequences of ITS, *GAPDH*, *ACT*, *TUB2*, and *CHS-1* sequences isolates of the *C*. *gloeosporioides* and *C*. *orchidearum* species complex and the ITS, *GAPDH*, *ACT*, and *TUB2* sequences of the *C*. *magnum* species complex were concatenated in Mesquite v. 2.75 [27]. Phylogenetic trees were generated using the Markov Chain Monte Carlo (MCMC) algorithm with Bayesian probabilities using MrBayes v. 3.2.6 [28] for each sequence data set. The best nucleotide substitution model for Bayesian inference (BI) analysis was determined by MrModelTest v. 2.3 [29].

Two analyses of four MCMC chains were conducted to sample trees and posterior probabilities of the model parameter. Markov Chains were run for 1 × 10^6^ generations and a tree was sampled every 1000 generations. The generated 50% majority rule consensus trees from the Bayesian analyses were viewed in FigTree v 1.3.1 [30]. In addition, a maximum likelihood (ML) phylogenetic analysis was conducted with 1000 bootstrap replications using MEGA v.6 [26].

To delimit the phylogenetically related but ambiguous species, sequences were analyzed using the Genealogical Concordance Phylogenetic Species Recognition (GCPSR) model by performing a pairwise homoplasy index (Φw, PHI) test, as recommended by Quaedvlieg et al. [31]. Four-locus concatenated sequences dataset (ITS, *TUB2*, *GAPDH*, and *ACT*) of closely related species were used for PHI test in Splits Tree 4 to determine the recombination level [17].

### Morphological analysis

The methods described by Kim et al. [15] were followed for morphological and cultural characterization of representative isolates from each *Colletotrichum* species identified in this study. The culture diameter measurements and appearance were recorded after seven days of growth at 25°C in the dark. The conidia were taken from the conidial ooze and mounted on a glass slide in lactic acid to analyze size and shape. At least 30 conidia were measured using an Olympus BX43 microscope with a magnification of 400×. The range of measurement was presented in the form of minimum-maximum (mean ± STD). To enhance the appressoria formation, the mounted slides were placed in a plastic box containing moistened tissue and incubated at 25°C in the dark. After three days of incubation, the size of 30 conidial appressoria formed at the end of the germ tube was measured as we as the conidia itself. The sizes of the conidia were analyzed with the statistical programme “R” v. 3.6.3 (R Development Core Team 2011). The R ggplot2 package was used to generate graphical plots [32]. To assess the temperature effect on mycelial growth, a 5-mm-diameter colony plug from the margin of a seven-day-old culture was placed in the center of a 90-mm-diameter fresh PDA plate. Three replicated PDA plates of each isolate were incubated at 10°C, 15°C, 18°C, 20°C, 22°C, 25°C, 30°C, 32°C, and 35°C in the dark. The colony diameter was measured every day for seven days. The optimum temperature for the mycelial growth rate was determined using SigmaPlot14 (Systat Software, San Jose, CA), based on the Gaussian method (4 parameter) for a non-linear regression analysis. Similarly, three replicates of each isolate were incubated at 25°C in the dark on PDA, oatmeal agar (OA), Difco, MI, USA), 72.5 g/L), malt extract agar (MEA) (Difco, MI, USA), synthetic low-nutrient agar (SNA) (Difco, MI, USA), and cornmeal agar (CA) (Difco, MI, USA), to determine the effect of these media on mycelial growth.

### Prevalence

The prevalence of *Colletotrichum* species in the sampled locations was estimated. The Isolation Rate (RI) was calculated using the formula described by Fu et al. [17]:

$$RI=(\frac{NS}{NI})\times 100,$$ (i)

Where NS is the number of isolates of a specific species and NI is the total number of isolates from each sample collected from a specific location. The overall RI was calculated considering NI as the total number of isolates obtained from OLA.

### Pathogenicity test

The pathogenicity of representative *Colletotrichum* isolates was determined on detached OLA leaves. Healthy leaves were collected, their surface sterilized with 70% ethanol, washed thrice with sterile distilled water, and then air dried on a sterilized tissue paper. Twenty leaves per isolate were inoculated using wound/drop and non-wound/drop inoculation methods [17]. For the preparation of the conidial suspensions, 15-days-old culture was flooded with sterile distilled water, filtered through sterile Kimtech tissue paper, and concentrated to 1 × 10^6^ conidia/mL using a hemocytometer. In the wound/drop method, the spore suspensions/water were dropped on the wounds. Leaves were wounded by pin-pricking both sides of the midrib (left and right) with a sterilized needle. Thereafter, 10 μL conidial suspensions was dropped on the left side, while sterile water was placed as a control on the right side of the same leaf. For the non-wound/drop, unwounded leaves were inoculated in the same way as described above. After inoculation, the leaves were placed into a plastic box containing moist tissue, covered with plastic film to maintain >80% relative humidity, and incubated at 25°C with a 12/12 h light/dark photoperiod. Symptom development on leaves was examined every day. The infection rate was determined as the proportion of infected leaves which presented typical anthracnose symptoms. Under wounded conditions, the symptom development was evaluated by determining the lesion size (diameter). Fungal colonies were re-isolated from the lesions of inoculated leaves and checked for morphological characteristic, in order to fulfill Koch’s postulates.

## Results

### Collected *Colletotrichum isolates* associated with OLA anthracnose

**Fig 3:**
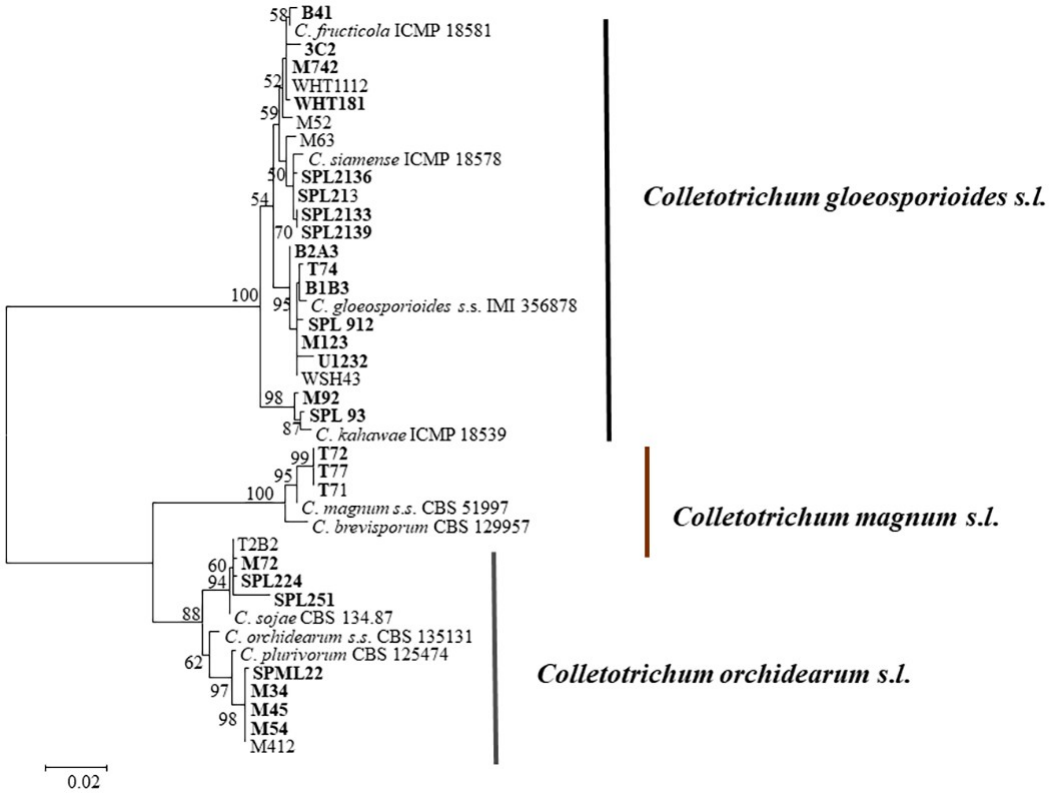
A maximum likelihood phylogenetic tree based on ITS, and *TUB2* sequences from 32 isolates from OLA and representative isolates of the different *Colletotrichum* species complexes. Bootstrap values ≥ 50% are shown at the nodes. The isolates selected for further phylogenetic analysis are shown in bold. The scale bar indicates 0.02 substitutions per site.

A total of 32 isolates of *Colletotrichum* spp. were collected from 30 OLA leaves affected by anthracnose in main OLA growing regions in South Korea, 19 isolates from Mungyeong and 13 from Sangju (Table 1). All isolates were initially identified based on its morphological characteristics and phylogenetic analysis using IT’S and TUB sequences (Fig 3). The isolates were preliminarily allocated to the following species complexes: 20 isolates belong to the *C*. *gloeosporioides*, nine isolates belong to *C*. *orchidearum* and three belong to *C*. *magnum* species complex (Fig 3). A total of 16 representative isolates from *C*. *gloeosporioides*, seven from *C*. *orchidearum* and three from *C*. *magnum* species complex were chosen for further analysis (Fig 3).

#### Multi-locus phylogeny

**Fig 4:**
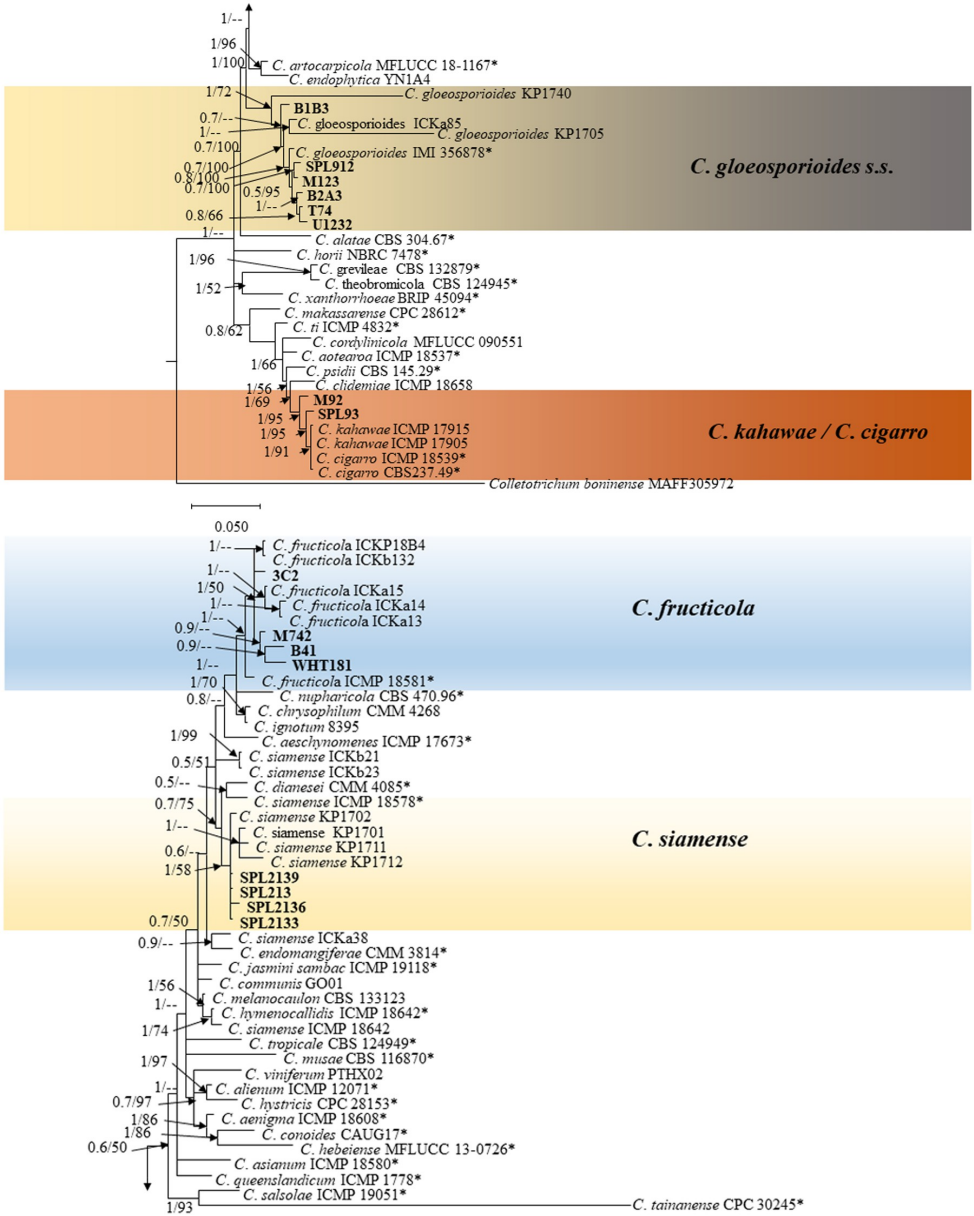
A Bayesian inference phylogenetic tree (50% majority consensus) of 54 isolates of the *C*. *gloeosporioides* species complex. The tree is rooted with *C*. *boninense* (MAFF305972). The tree was constructed using a concatenated data set of ITS, *ACT*, *TUB2*, *CHS-1*, and *GAPDH* sequences. Bayesian posterior probability (PP ≥ 0.50) and maximum likelihood bootstrap support values (ML ≥ 50) are shown at the nodes. *Indicates the ex-type strains. The isolates collected in this study are indicated in bold. Colored blocks indicate clades containing isolates from *Atractylodes ovata* in this study. The scale bar indicates 0.2 expected changes per site.

*Colletotrichum gloeosporioides complex*. The phylogram in Fig 4 shows the relationships amongst the isolates in the *C*. *gloeosporioides* species complex. The combined data set consisted of ITS, *GAPDH*, *ACT*, *TUB2*, and *CHS-1* sequences of 75 isolates with *Colletotrichum boninense* (MAFF305972) as out-group and 1951 characters including alignment gaps. The boundaries of genes within the concatenated alignment were: ITS: 1–581, *TUB2*: 582–1036, *GAPDH*: 1037–1385, *CHS-1*: 1386–1691, *ACT*: 1692–1951. A total of 15002 trees were used for calculating posterior probabilities. The topology of the ML tree complemented the topology of the BI tree. Posterior probabilities (> 0.5) and bootstrap support values (> 50%) were plotted onto the nodes (BI/ML). Phylogenetic analysis of *C*. *gloeosporioides* species complex showed that present isolates from OLA anthracnose clearly clustered in four clades: four isolates clustered with ex-type *C*. *fructicola* (1/50), four isolates clustered with ex-type *C*. *siamense* (0.5/51), six clustered with ex-type *C*. *gloeosporioides* s.s (1/72), while the remaining two isolates formed a distinct clade with ex-type *C*. *kahawae* and *C*. *cigarro* (1/95). The *C*. *siamense* isolates were further confirmed by phylogenetic analysis using *ApMat* sequence (S1 Fig). The *C*. *kahawae* /*C*. *cigarro* isolates are also subjected to phylogenetic analysis based on concatenated sequences of ITS and *ApMat* to compare with strains of both species (*C*. *kahawae* and *C*. *cigarro*). The reference isolates of *C*. *kahawae* and *C*. *cigarro* were adopted from the study conducted by Cabral et al. [33]. Phylogenetic tree showed that present isolates were clustered together with *C*. *cigarro* strains (S2 Fig).

**Fig 5:**
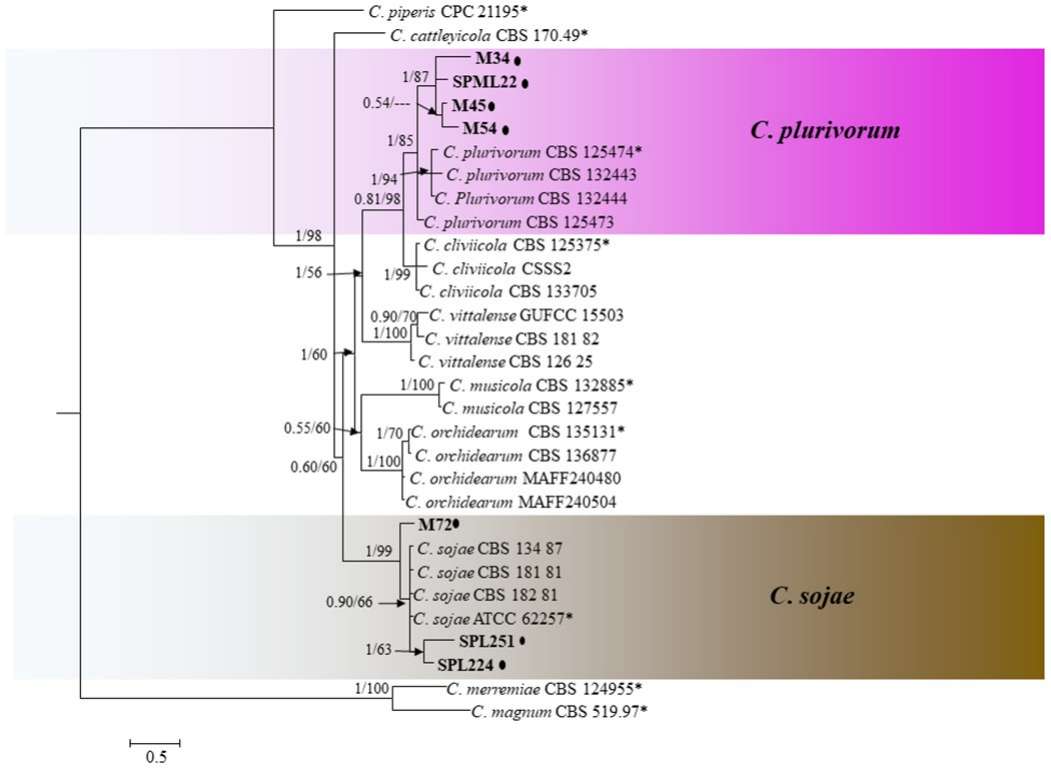
A Bayesian inference phylogenetic tree (50% majority consensus) of 31 isolates in the *C*. *orchidearum* species complex. The tree is rooted with *C*. *merremiae* (CBS 124955) and *C*. *magnum* (CBS 51997). The tree was constructed using a concatenated data set of ITS, *ACT*, *TUB2*, *CHS-1* and *GAPDH* sequences. Bayesian posterior probability (PP ≥ 0.50) and maximum likelihood bootstrap support values (ML ≥ 50) are shown at the nodes. *Indicates the ex-type strains. The isolates collected in this study are indicated in bold. Coloured blocks indicate clades containing isolates from *Atractylodes ovata* in this study. The scale bar indicates 0.5 expected changes per site.

*Colletotrichum orchidearum complex*. Five loci (ITS, *GAPDH*, *ACT*, *CHS-1*, and *TUB2*) were used for phylogenetic analysis of the *C*. *orchidearum* species complex. The combined data set consisted of 31 isolates sequences with *C*. *magnum* (CBS 519 97) and *C*. *merremiae* (CBS 124955) as the out-group and 1780 characters including alignment gaps (Fig 5). The boundaries of the gene in the alignment were ITS: 1–548, *TUB2*: 549–995, *GAPDH*: 996–1249, *ACT*: 1250–1505, *CHS-1*: 1506–1780. A total of 14,720 credible trees were used to estimate posterior probabilities. Posterior probabilities of BI bootstrap support values of ML analysis were given at the nodes (BI/ML), which were congruent between the Bayesian phylogeny and the ML tree. The result showed that isolates from infected OLA in the *C*. *orchidearum* species complex clustered into two clades (Fig 5): three isolates with the ex-type *C*. *sojae* isolates (1/99) and four with the ex-type *C*. *plurivorum* isolates (1/85).

**Fig 6:**
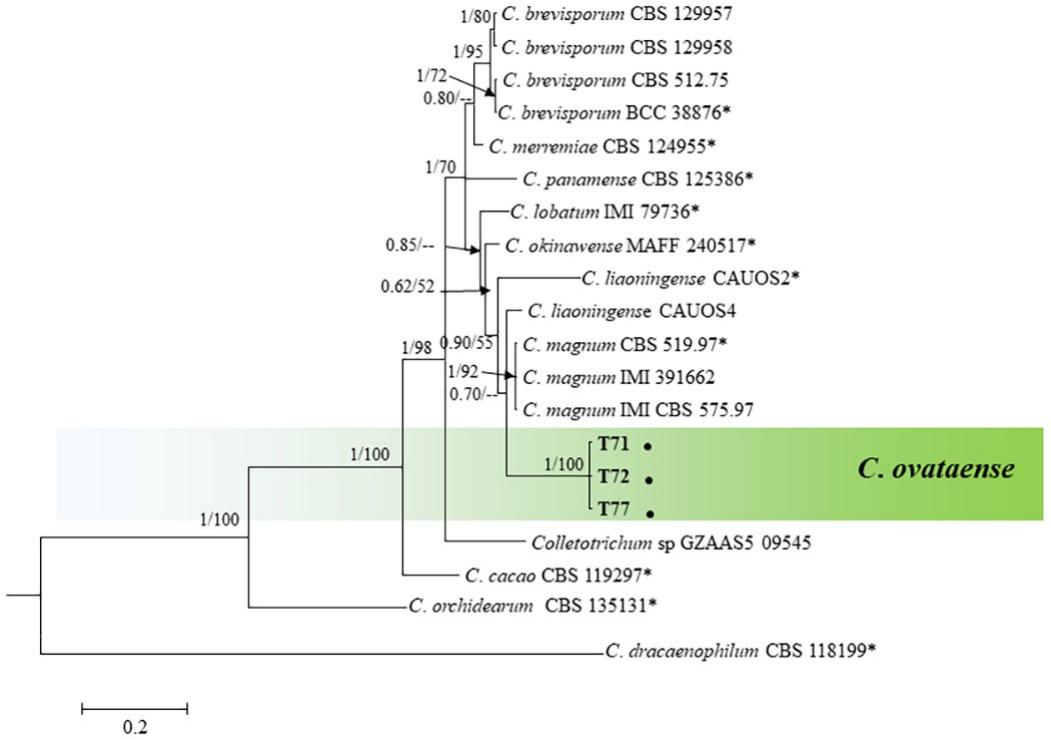
A Bayesian inference phylogenetic tree (50% majority consensus) of 31 isolates in the *C*. *magnum* species complex. The tree was constructed using a concatenated data set of ITS, *ACT*, *TUB2*, and *GAPDH* sequences. Bayesian posterior probability (PP ≥ 0.50) and maximum likelihood bootstrap support values (ML ≥ 50) are shown at the nodes. The isolates collected in this study are indicated in bold. *Indicates the ex-type strains. Coloured blocks indicate clades containing isolates from *Atractylodes ovata* in this study. The scale bar indicates 0.2 expected changes per site. *Colletotrichum orchidearum* (CBS 135131) and *C*. *dracaenophilum* (CBS 118199) were used as out-group.

*Colletotrichum magnum complex*. The phylogenetic tree was constructed for the *C*. *magnum* species complex using concatenated sequences of ITS, *GAPDH*, *ACT* and *TUB2* (Fig 6). The combined aligned sequences data consisted of 25 isolates and 1555 characters including alignment gaps. In this case, representative isolates form *C*. *orchidearum s*.*l*. and *C*. *dracaenophilum* species complex [18] were used as out-group. The boundaries of the gene in alignment were ITS: 1–513, *TUB2*: 514–943, *GAPDH*: 944–1274, *ACT*: 1275–1555. Posterior probabilities were estimated from a set of 3097 credible trees. The topology of ML tree complemented the topology of the BI tree. Posterior probability (>0.5) and bootstrap support value (>50%) were plotted on nodes (BI/ML). The phylogenetic tree showed that thee isolates formed a clad themselves with very high support (1/ 100) (Fig 6).

**Fig 7:**
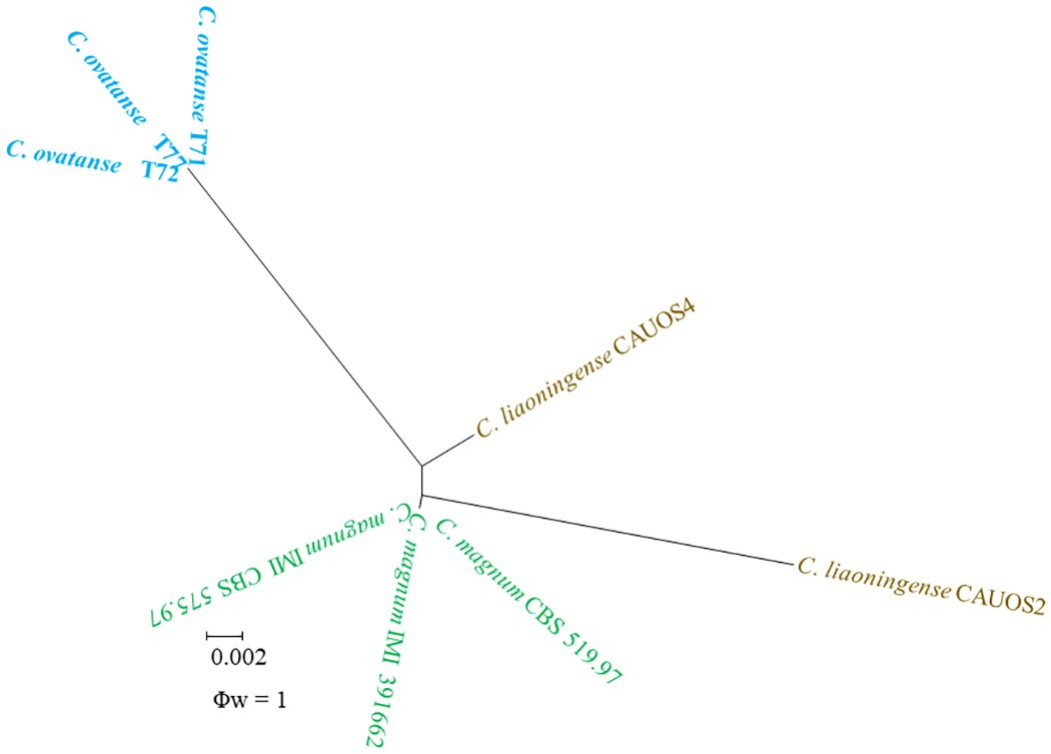
The result of the pairwise homoplasy index (PHI) tests of closely related species using both LogDet transformation and splits decomposition. Resulting PHI test value (Φw) < 0.05 indicate significant recombination within the dataset.

The concatenated sequences dataset of ITS, *GAPDH*, *ACT*, and *TUB2* were subjected to a PHI test to estimate the recombination level within phylogenetically related species. The result of a PHI test was above the 0.05 threshold (Φw = 1), this indicated no significant recombination in the dataset. So, there were no significant recombination events observed between *C*. *ovataense* and phylogenetically-related isolates or species (*C*. *magnum s*.*s*. isolate CBS 519 97, IMI 391662, and CBS 575 97 and *C*. *liaoningense* isolate CAUOS2 and CAUOS4) (Fig 7).

#### Morphological analysis

*Taxonomy*. The new species identified in this study are described below. Previously reported species were not described in detail in this study.

**Fig 8:**
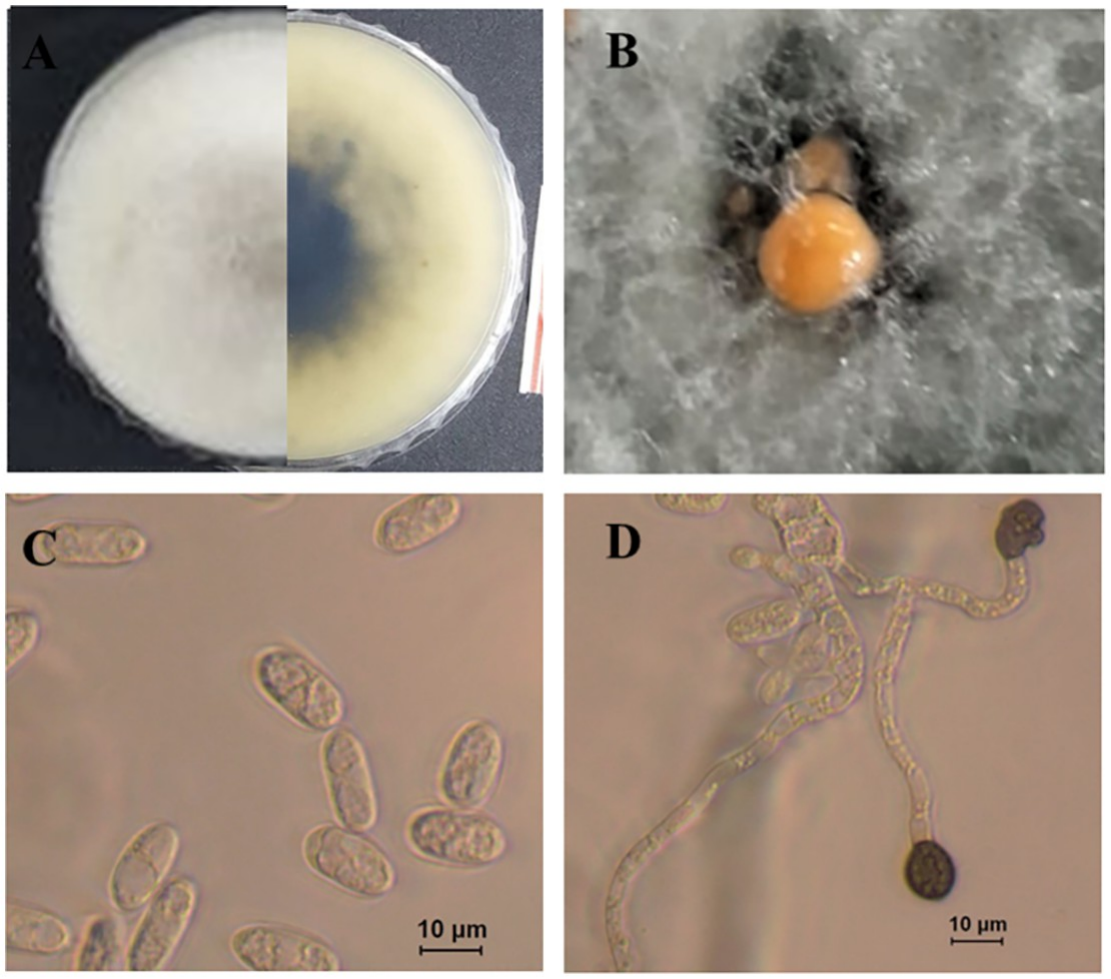
Morphological characteristics of *C*. *cigarro* (isolate SPL93). A: Colony on PDA above and below. B: Conidioma. C: Conidia. D: Appressoria.

***Colletotrichum cigarro*** (B.S. Weir & P.R. Johnst.) A. Cabral & P. Talhinhas, Plants. 9: 502. 2020. (Fig 8).

Morphological description and Illustration: see Weir et al. [34].

Culture examined: South Korea, Gyeongbuk Province, Mungyeong, isolated from leaf lesions of *Atractylodes ovata* De Candolle, 20 June 2019, B. B. N. D. Romain, Ex-type culture SPL93 = KACC49842. Gyeongbuk Province, Sangju, on leaves of *Atractylodes ovata* e, 25 June 2020, B. B. N. D. Romain (Culture M92).

Notes: There are two subspecies of *C*. *kahawae sensu* Waller *et al*. [35] namely: *C*. *kahawae* subsp. *kahawae* and *C*. *kahawae* subsp. *cigarro* [34]. *Colletotrichum kahawae* subsp. *cigarro* has been erected as new species (*C*. *cigarro*) recently Cabral et al. [33]. In this study we have isolated *C*. *cigarro* from OLA and identified it based on morphological and molecular characteristics.

**Fig 9:**
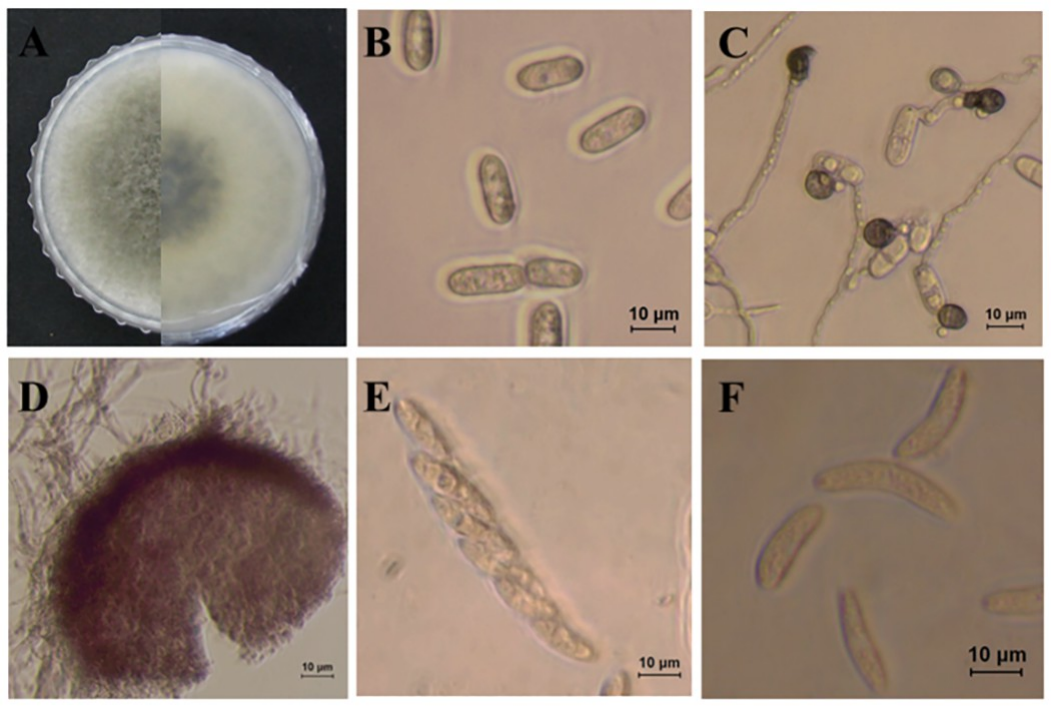
Morphological characteristics of *C*. *fructicola* (isolate 3C2). A: Colony on PDA above and below. B: Conidia. C: Appressoria D: Perithecium. E: Ascus. F: Ascospores.

***Colletotrichum fructicola*** Prihastuti, L. Cai and K.D. Hyde, Fungal Divers. 39: 158. 2009. (Fig 9).

Morphological description and Illustration: Prihastuti et al. [36].

Culture examined: South Korea, Gyeongbuk Province, Mungyong, isolated form leaf lesion of *Atractylodes ovata*, 25 June. 2019, B. B. N. D. Romain. Ex-type culture 3C2 = KACC49840 (culture = B41, M742, and WHT181).

Notes: Prihastuti et al. [36] first described *C*. *fructicola* from coffee berries in Thailand and subsequently reported it as the causal agent of anthracnose on several plants including Asian pear, pear, and mandarin orange [17, 34]. In South Korea, this species was identified as the causal agent of this disease in apple, peach, and grape [15, 16, 37].

**Fig 10:**
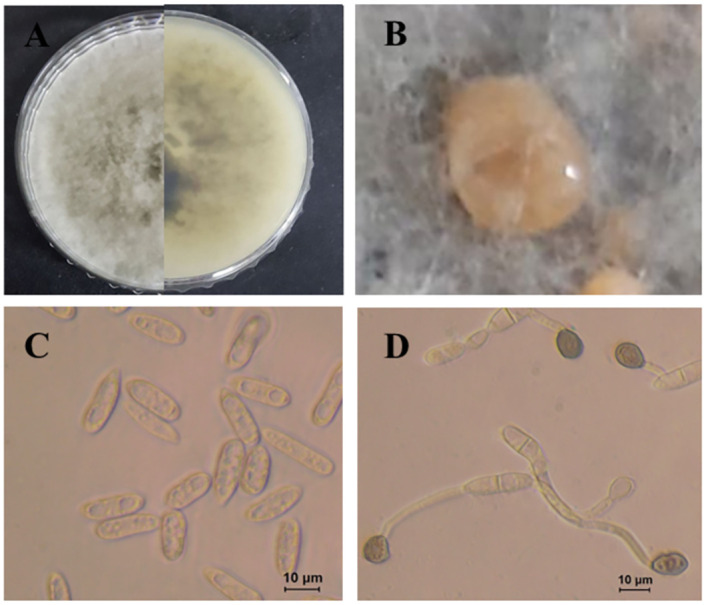
Morphological characteristics of *C*. *gloeosporioides s*.*s*. (isolate SPL912). A: Colony on PDA above and below. B: Conidioma. C: Conidia. D: Appressoria.

***Colletotrichum gloeosporioides*** (Penz.) Penz. & Sacc., Atti Reale Ist. Veneto Sci. Lett. Arti., Serie 6, 2: 670. 1884. (Fig 10).

Morphological description and illustration: Cannon et al. [38] and Weir et al. [34].

Culture examined: South Korea, Gyeongbuk Province, Sangju, isolated from leaf lesions of *Atractylodes ovata*, 20 June 2019, B. B. N. D. Romain. Ex-type culture SPL912 = KACC49841. Gyeongbuk Province, Mngyeong, on leaves of *Atractylodes ovata* De Candolle, 25 June 2020, B. B. N. D. Romain (Culture B2A3, M123, T74, U1232, B1B3).

Notes: *Colletotrichum gloeosporioides s*.*s*. has already been reported to be responsible of OLA anthracnose in Korea [4] although these previous identifications were only based on morphological characteristics and therefore unreliable. In this study, six isolates from OLA were identified as *C*. *gloeosporioides* using both morphological and multi-loci molecular analysis, confirm as anthracnose pathogen of OLA. *Colletotrichum gloeosporioides* has been also reported from common fruits including apple, plume, and persimmon in Korea [15, 16, 19, 20].

***Colletotrichum ovataense*** O. Hassan, J. S. Kim, B. B. N. D. Romain & T. Chang, sp. nov.

**Fig 11:**
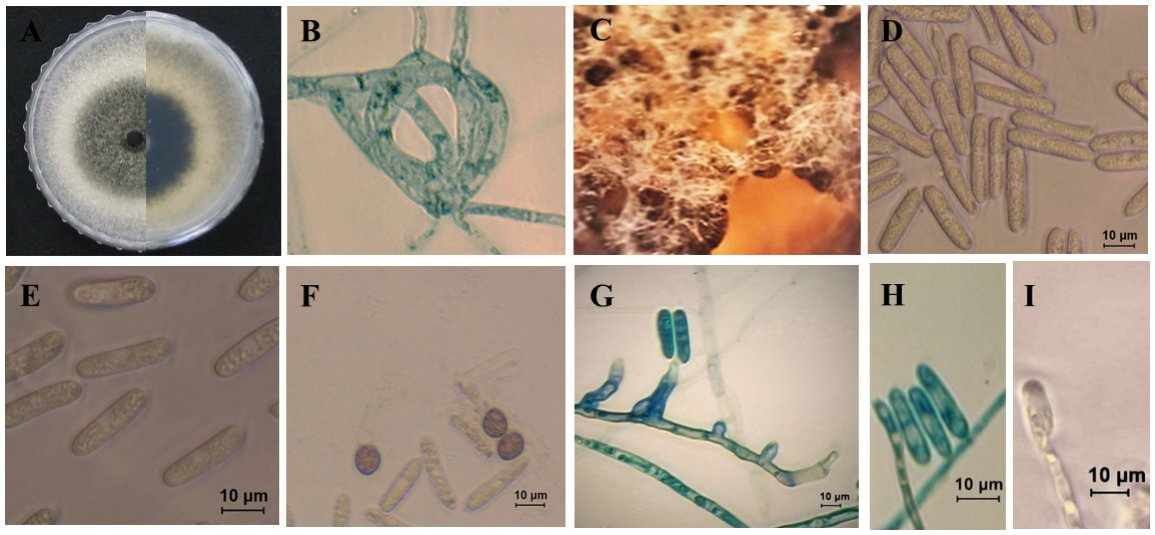
Morphological characteristics of *C*. *ovetaense* (isolate T72). A: Colony on PDA above and below. B: Hyphal coil C: Conidioma. D-E: Conidia. F: Appressoria. G-I: Conidiophores.

MycoBank MB839114; Fig 11.

*Etymology*: Referring to the host species (*A*. *ovata*) from which the fungus was isolated.

Morphological descriptions: *Teleomorph* was not observed.

*Anamorph on PDA* (KACC 49789). Vegetative hyphae branched, hyaline, smooth-walled, septate, and with 1.8–8.6 μm diam, sometimes forming coils. Spore masses were orange. Setae not observed. Conidiophores hyaline, unbranched, 1–4 septate and 15–96 × 3–7 μm. Conidiogenous cells hyaline, smooth-walled, subcylindrical, 17.5–31 × 4.5–7. μm, opening 1–2.0 μm diam, collarette 1.5 μm long. *Conidia* hyaline, smooth-walled, aseptate, straight, cylindrical with both ends round or with one end slightly acute, 20.5–30 × 5–8 μm (mean ± SD = 25.6 ± 2.5 × 6.4 ± 0.6 μm), L/W ratio = 4.0. Appressoria mostly single, dark brown, smooth-walled, globose, the edge entire 8–13.5× 7.5–11.5 μm (mean ± SD = 10.8 ± 1.4 × 9. ± 1.0 μm), L/W ratio = 1.8.

*Anamorph on SNA* (KACC 49789). Conidiomata not observed, conidia formed directly on hyphae, conidiophores hyaline, smooth-walled, septate, unbranched, on average 49 μm long. Conidiogenous cells hyaline, smooth-walled, cylindrical, 16.5–30.5 × 4–6.5 μm (mean ± SD = 25.8 ± 2.2 × 5.6 ± 0.9 μm), opening 1–1.5 μm diam, periclinal thickening inconspicuous. *Conidia* hyaline, smooth-walled, aseptate, straight, cylindrical with both ends round or one end round and one end acute, 18.5–26 × 4.5–7.0 μm (mean ± SD = 22.5 ± 1.8 × 5.5 ± 0.7 μm), L/W ratio = 3.7.

*Appressoria* (very few observed), mostly single, pale to medium brown, smooth-walled, subglobose to globose, the edge entire, 8.5–14 × 7.5–12 μm (mean ± SD = 11.7 ± 1.5 × 8.8 ± 1.1), L/W ratio = 1.3.

***Culture characteristics***: Colonies on PDA white-gray with black zonation at the center; reverse creamy white with black zonation at center. The diameter of the colony was of 58.3 mm after seven days growing at the rate of 8.3 mm day^−1^. Colonies on SNA cottony, white, with white aerial mycelium, and covered the filter paper, grew at a rate of 5 mm day^−1^. Colonies on OA presented a growth rate of 7.8 mm day^−1^, a surface white-gray and entire margins. Colonies on MEA white, and a pale grey flat surfaces with white margins. Colonies on CA submerged with sparse mycelium and grew 59.7 mm in seven days. Colony on V8 white, flat, and grew at a rate of 5.5 mm day^−1^.

***Culture examined***: South Korea, Gyeongbuk Province, Mungyeong City on leaves of *Atractylodes ovata*, 25 June, 2020, B. B. N. D. Romain (Ex-type KACC 49789, culture T72); ibid. T71 and T77.

Notes: The strains of new species were isolated from infected leaves of OLA from a commercial field in Mungyeong, South Korea. The multi-locus (ITS, *GAPDH*, *ACT* and *TUB2*) phylogenetic tree analysis revealed that the isolates of *C*. *ovataense* is closely related to *C*. *magnum* s.s. (isolates; CBS 519.97, IMI 391662 and CBS 575.97) (Fig 6). *Colletotrichum magnum* was reported as the causal agent of anthracnose of cucurbits in the USA, *Lobelia chinensis* in china, and *Carica papaya* in Brazil [18].

The results of BLASTn search showed that they (*C*. *ovataense* isolate T72 and *C*. *magnum* s.s. isolate CBS 519.97) are different in *GAPDH* (90% identity, 24 bp differences), *TUB* (98.5%, 5bp), ITS (99.8%, 1bp) and *ACT* (100%, 0 bp).

In morphology, the conidia (18.5–26 × 4.5–7.0 μm) and appressoria (8.5–14 × 7.5–12 μm) of *C*. *ovataense* are bigger than those of *C*. *magnum* s.s. (conidia: 15.5–19 × 4–4.5 μm; appressoia: 6.5–12.5 × 4.5–7.5 μm) (S3 Table).

**Fig 12:**
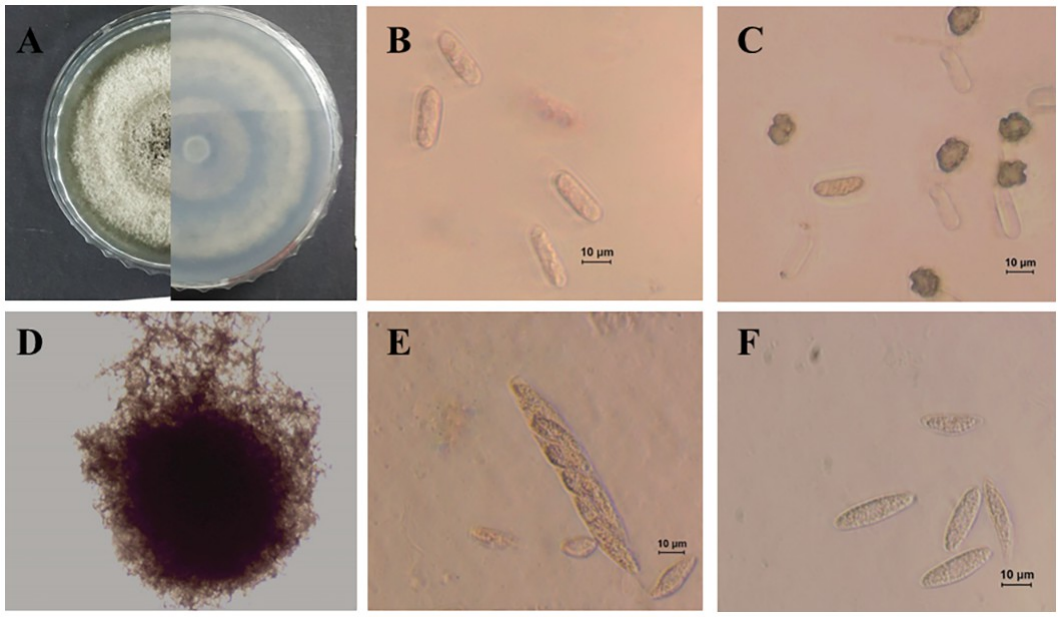
Morphological characteristics of *C*. *plurivorum* (isolate SPML22). A: Colony on PDA above and below. B: Conidia. C: Appressoria D: Perithecia. E: Ascus. F: Ascospore.

***Colletotrichum plurivorum*** Damm, Alizadeh & Toy, Stud. Mycol. 92: 1–46. 2019. (Fig 12).

Morphological description and Illustration: Damm et al. [18].

Culture examined: South Korea Gyeongbuk Province, Sangju, on leaves of *Atractylodes ovata*, 20 June. 2019, B. B. N. D. Romain, Ex-type culture SPML22 = KACC49843. Gyeongbuk Province, Mungyong, on leaves of *Atractylodes ovata*, 25 June, 2020, B. B. N. D. Romain (Culture, M34, M45, M54).

Notes: *Colletotrichum plurivorum* (synonym: *C*. *sichuanensis*) has a large host range including *Phaseolus lunatus*, *Gossypium* spp., Spathiphyllum wallisii, *Phaseolus vulgaris*, and *Coffea* spp. [18]. In this study, four isolates were identified based on morphological characteristics and multigene phylogenetic analysis.

**Fig 13:**
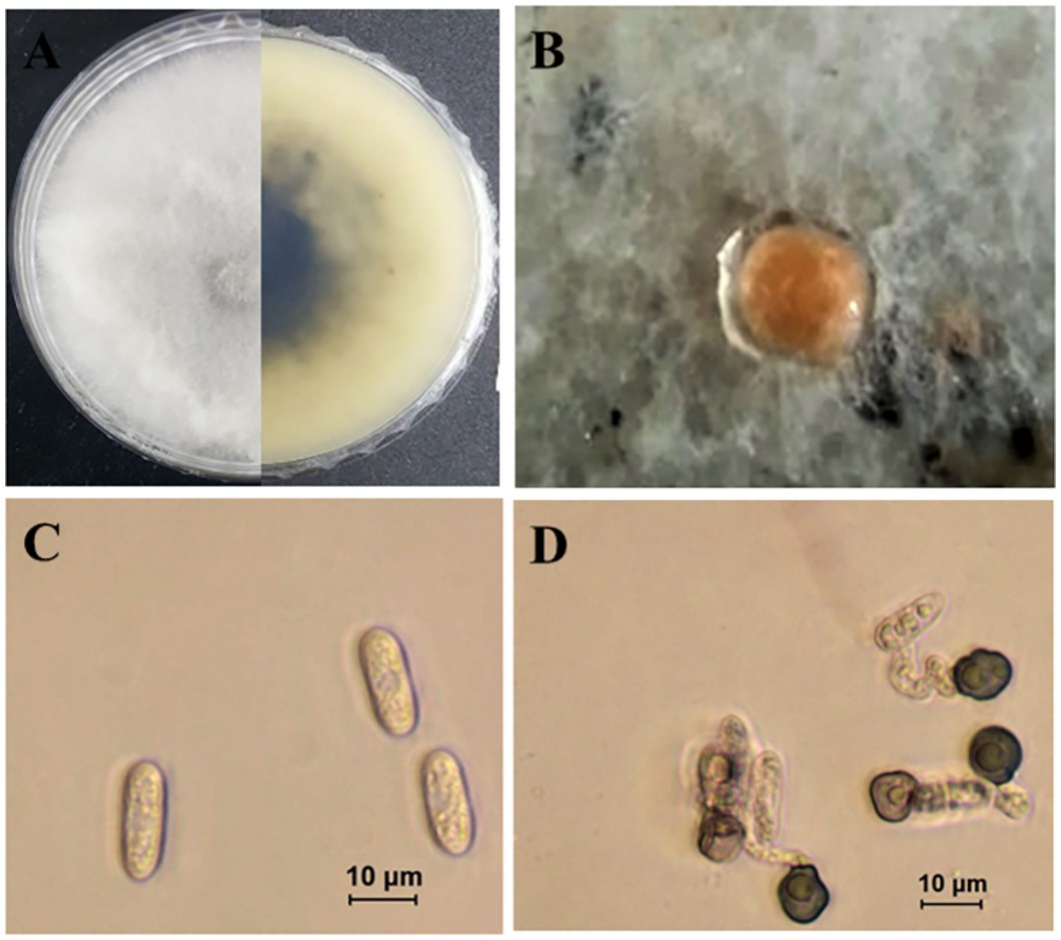
Morphological characteristics of *C*. *siamense* (isolate SPL2136). A: Colony on PDA above and below. B: Conidioma. C: Conidia. D: Appressoria.

***Colletotrichum siamense*** Prihastuti, L. Cai and K.D. Hyde, Fungal Diversity 39: 158. 2009. (Fig 13).

Morphological description and Illustration: Prihastuti et al. [36]

Culture examined: South Korea, Gyeongbuk Province, Sangju on leaves of *Atractylodes ovata*, 20 June. 2019, B. B. N. D. Romain, Ex-type culture SPL213 = KACC49843 (Culture, SPL2133, SPL2136, SPL2139).

Notes: *Colletotrichum siamense* is a cosmopolitan pathogen with a widehost range among the species within the *C*. *gloeosporioides* species complex [15, 16, 19, 20, 39–41]. It was described by Prihastuti et al. [36] from coffee berries in Thailand. Sharma et al. [40] regarded *C*. *siamense* as a species complex, however Liu et al. [41] verified it to be a single species. In this study, four isolates were confirmed as *C*. *siamense* based on morphological characteristics and multigene molecular analysis. *C*. *siamense* was reported as the causal agent of anthracnose of many crops in South Korea, including persimmon, apple, plume, and peach [15, 16, 19, 20].

**Fig 14:**
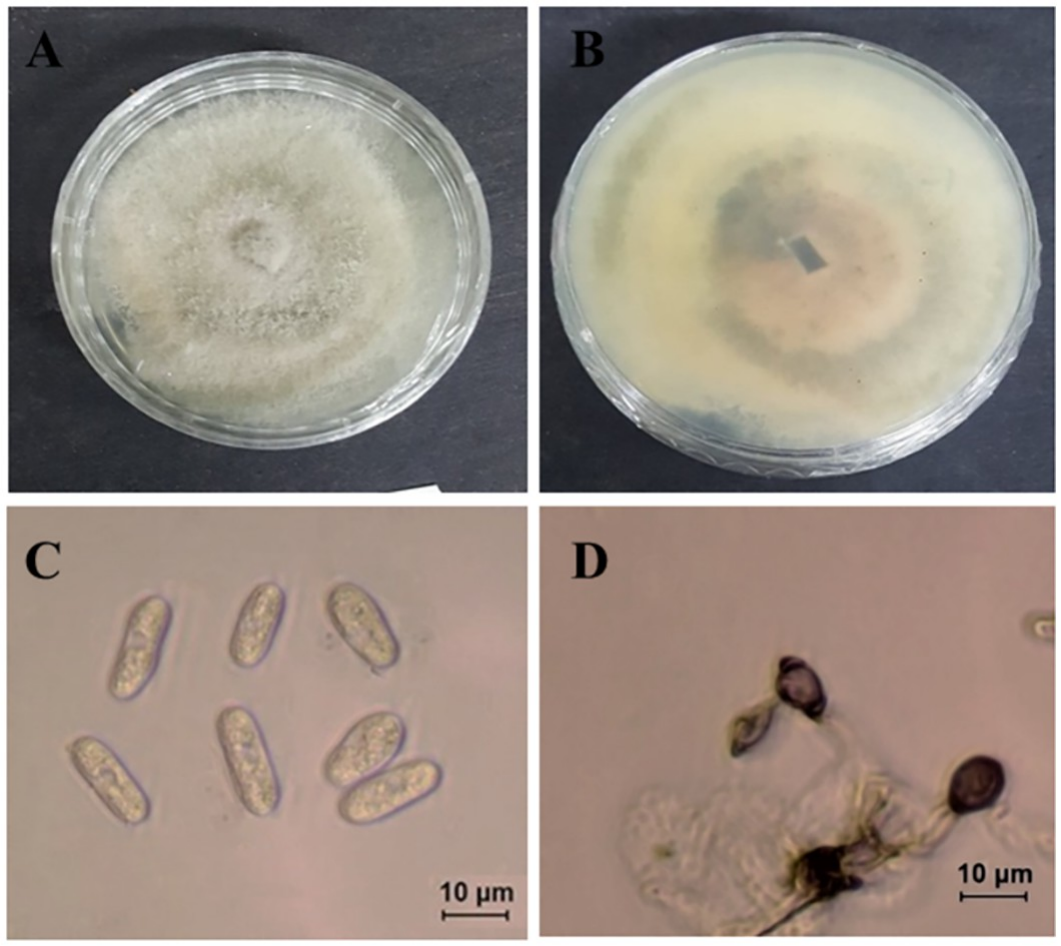
Morphological characteristics of *C*. *sojae* (isolate M72). A-B: Colony on PDA above and below. C: Conidia. D: Appressoria.

***Colletotrichum sojae*** Damm& Alizadeh, Stud. Mycol. 92: 1–46. 2019. (Fig 14).

Morphological description and Illustration: Damm et al. [18].

Culture examined: South Korea Gyeongbuk Province, Sangju, on leaves of *Atractylodes ovata* De Candolle, 20 June, 2019, B. B. N. D. Romain, Ex-type culture SPL251 = KACC49845 (Culture SPL224) Gyeongbuk Province, Mungyong, on leaves of *Atractylodes ovata* De Candolle, 25 June, 2020, B. B. N. D. Romain (Culture M72).

Notes: Strain ATCC 62257 was isolated from *Glycine max* and had originally been identified as *Glomerella glycines*, which was revealed to be a synonym of *C*. *truncatum*. Damm et al. [18] found that the morphological characteristics of this strain are not similar to that of *C*. *truncatum*, consequently being described as a new species, *C*. *sojae*. This fungal species has been reported as the causative agent of anthracnose of many crops including *Fabaceae* (Glycine, *Medicago*, *Phaseolus*, *Vigna*), but also on *Amaranthaceae* (*Amaranthus*), *Asteraceae* (*Arctium*), *Solanaceae* (*Capsicum*), and *Orchidaceae* (*Bletilla*) [42].

**Fig 15:**
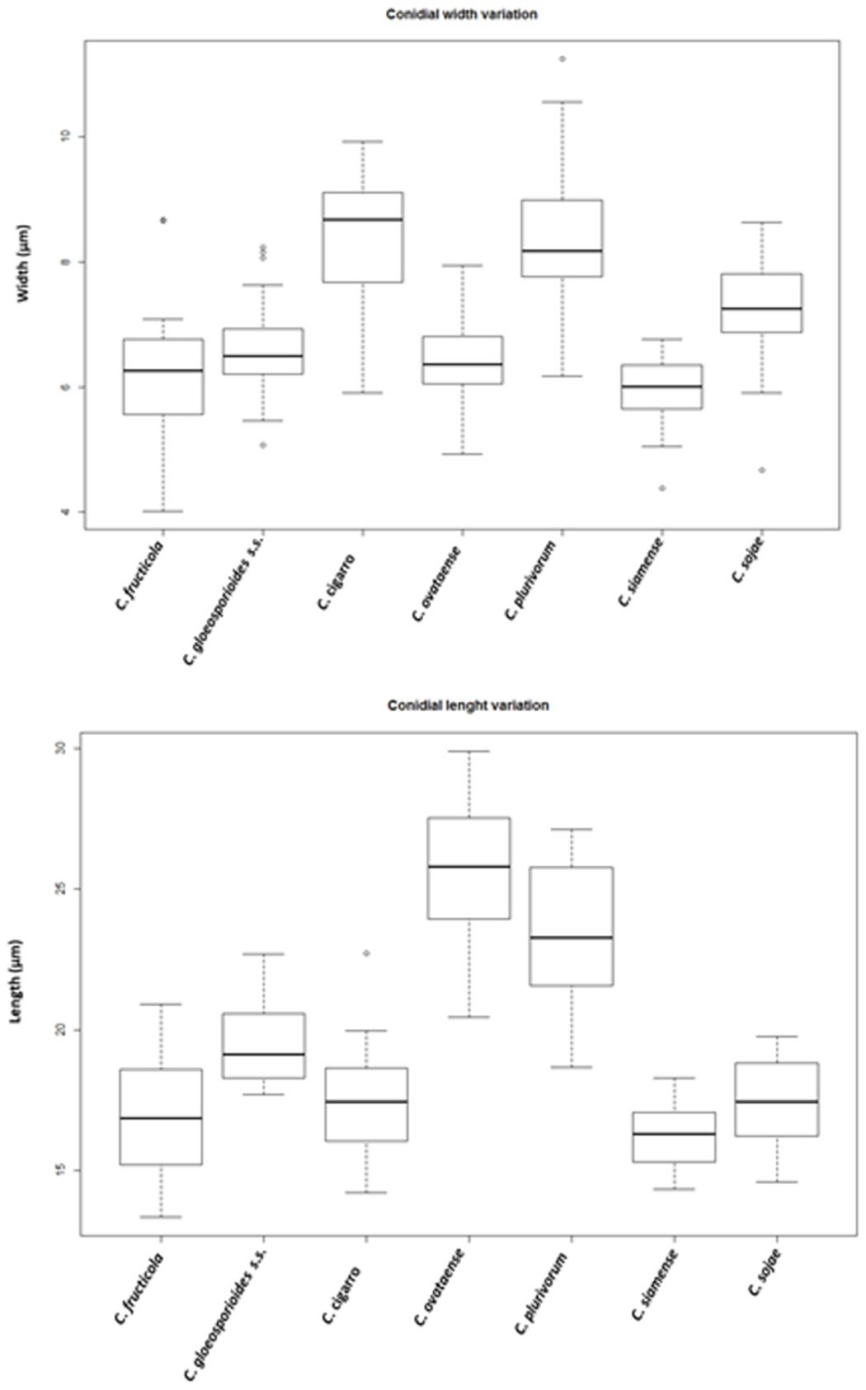
Box plots showing the variation in length and width of conidia produced in PDA.

Based on morphological characteristics and multigene phylogenetic analysis, the 20 isolates were assigned to seven *Colletotrichum* spp. as the cause of anthracnose in OLA. Amongst the seven species from OLA, *C*. *gloeosporioides s*.s. was previously reported species, five species were reported for the first time and one described as new species in this study. Colony and conidia characters of all seven *Colletotrichum* species were variable between different species (Table 2 and Fig 15). The effect of media on mycelium growth was summarized in Table 2. While all the species grew well in MEA followed by PDA, the same species grew differently on different culture media. For example, *C*. *gloeosporioides s*.s. grew faster in MEA and *C*. *sojae* in PDA. *C*. *fructicola* and *C*. *plurivorum* were characterized in part by the development of sexual morphs in culture.

**Table 2:** Growth of *Colletotrichum* spp. on different culture media after seven days.

Species	Mycelial growth on different culture media (mean ± SD; diameter = mm)			
	PDA^y^	OA	CA	MEA
*C*. *fructicola* (strain 3C2)	59.6 ± 4.5	42.4 ± 2.7	59.0 ± 5.4	60.4 ± 2.9
*C*. *gloeosporioides s*.s. (strain SPL912)	56.6 ± 3.5	49.1 ± 1.9	60.7 ± 2.5	65.9 ± 5.7
*C*. *cigarro* (strain SPL93)	58.4 ± 2.3	46.6 ± 3.2	53.6 ± 2.0	57.8 ± 3.4
*C*. *ovataense* (strain T72)	58.3 ± 3.8	55.1 ± 2.1	59.7 ± 3.5	54.9 ± 2.1
*C*. *siamense* (SPL2136)	37.3 ± 3.0	38.8 ± 1.6	54.7 ± 2.9	45.4 ± 1.5
*C*. *sojae* (M72)	63.8 ± 2.2	60.1 ± 3.46	59.2 ± 1.7	60.2 ± 2.5
*C*. *plurivorum* (strain SPML22)	62.7 ± 2.2	50.1 ± 3.0	53.9 ± 3.2	62.0 ± 2.8

^y^ PDA = potato dextrose agar; OA = oatmealagar; CA = cornmeal agar; MEA = malt extract agar

Conidial sizes of all the identified species are shown in Fig 15. There was some variation in the conidia of different species and size ranges often overlaped (Fig 15). *C*. *ovataense* produced the longest conidia, followed by *C*. *plurivorum*. The shape of approsoria between the different identified species was consistent with their little differences in the outline: some species presented a highly lobed outline, while this was not observed in other species.

**Fig 16:**
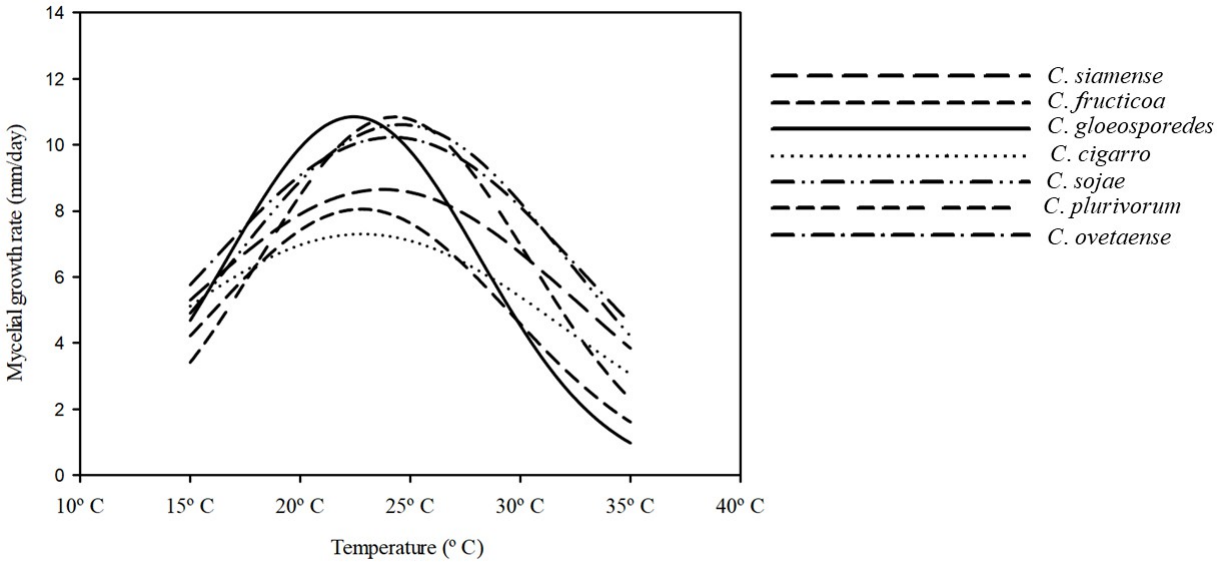
Effect of temperature on mycelium growth rates of *Colletotrichum* spp. different line type represent the mean growth rates of different species at the tested temperatures. Non-linear regression (Gaussian process) was used to determine the optimum temperature for mycelial growth.

The growth rate of the representative isolates on PDA between 15°C and 35°C is illustrated in Fig 16. The maximum growth rate of all *Colletotrichum* species was estimated to be between 22°C–25°C. For example, the maximum growth rate of *C*. *gloeosporioides* s.s. was estimated to be 22°C, this of *C*. *plurivorum* and *C*. *ovataense* was of 25°C.

**Fig 17:**
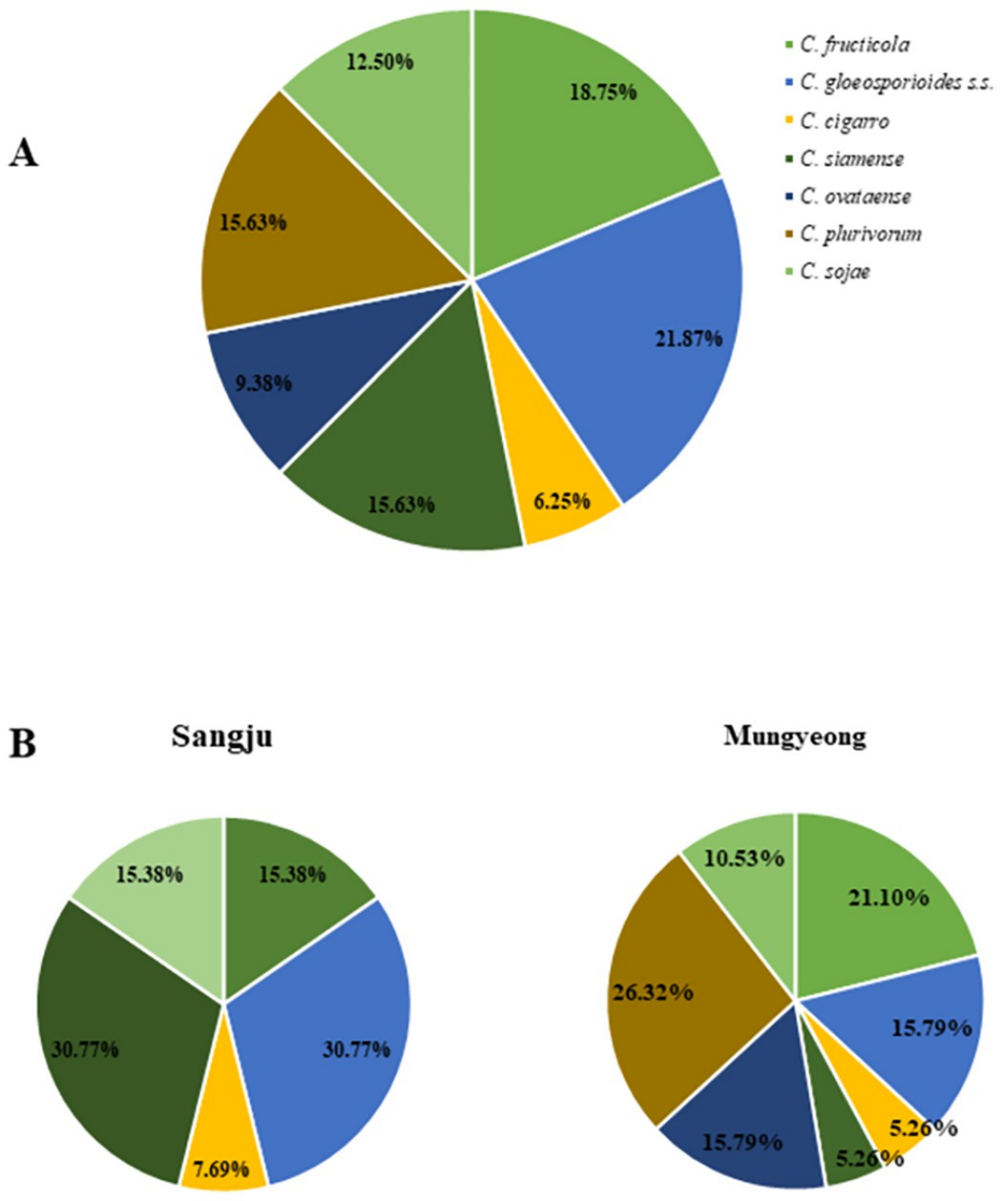
The prevalence of *Colletotrichum* species isolated from OLA. A: Overall isolation rate (%) of *Colletotrichum* species. B: isolation rate (%) of *Colletotrichum* species from each sampled area.

*Prevalence of colletotrichum species*. The result of prevalence analysis of seven *Colletotrichum* spp. showed that *C*. *gloeosporioides s*.s. was the most frequently isolated species (22.0%) from both sampling areas, followed by *C*. *fructicola* (18.0%), *C*. *siamense* (15.6%), *C*. *plurivorum* (15.6%), *C*. *sojae* (12.0%), *C*. *ovataense* (9.0%), and *C*. *cigarro* (6.0%) (Fig 17A).

The analysis of prevalence of seven *Colletotrichum* spp. isolates from each sampling areas revealed *C*. *gloeosporioides s*.s. and *C*. *siamense* as the most dominant species in Sangju, accounting for 31% of the obtained isolates. Isolates of other species of *Colletotrichum* spp. (except *C*. *plurivorum* and *C*. *ovataense*) accounted for less than 16% (Fig 17B). No isolates for *C*. *plurivorum* and *C*. *ovataense* were obtained from the Sangju area. Isolates obtained from Mungyeong region represented all seven species. In this case, the isolates of *C*. *plurivorum* represented a larger prevalence (26%) when compared to other species. The least prevalent isolates obtained in this region were *C*. *siamense* and *C*. *cigarro* (Fig 17B).

#### Pathogenicity test

**Fig 18:**
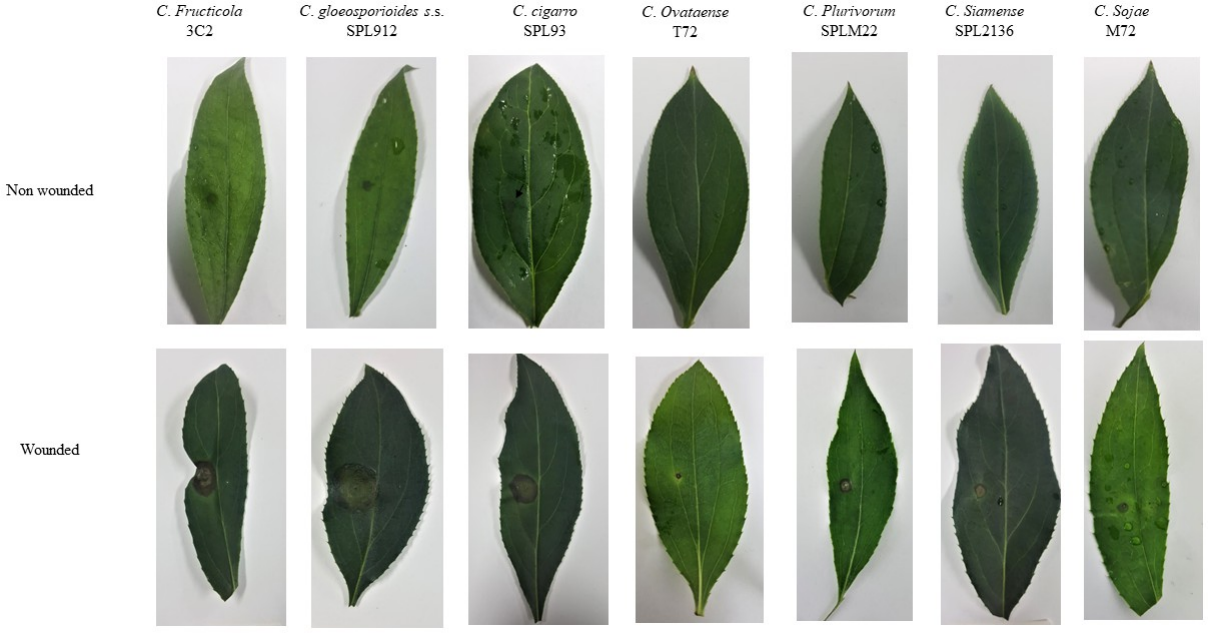
The symptoms of OLA leaves induced by *Colletotrichum* spp. with unwounded and wounded inoculation. The lesion on leaves were photographed seven days post inoculation. The left side of each leaflet was inoculated with 10 μl spore suspension (10^6^ spores/ml) and the right side with water (control).

To confirm Koch’s postulates for *C*. *fructicola* 3C2, *C*. *gloeosporioides s*.s. SPL912, *C*. *cigarro* SPL93, *C*. *ovataense* T72, *C*. *plurivorum* SPML22, *C*. *siamense* SPL2136 and *C*. *sojae* M72, its pathogenicity was tested on wounded and non-wounded detached leaves at 25°C. Under unwounded conditions, only *C*. *fructicola* 3C2, *C*. *gloeosporioides s*.s. SPL912 and *C*. *cigarro* SPL93 caused the necrotic lesion. The lesions were small (3–5 mm in diameter) and appeared 5 to 7 dpi (Fig 18).

These fungal species showed low infection rate (10%–30%) under unwounded condition, although all were pathogenic to leaves at various infectious rates, under wounded conditions (Table 3).

**Table 3:** Infection rates and disease incidence of *Colletotrichum* spp. inoculated on leaves of *Atractylodes ovata*.

Species	Strain	Infection rate		Disease incidence (%)	
		Non wounded	Wounded	Non wounded	Wounded
*C*. *fructicola*	3C2	2/20	20/20	10	100
*C*. *gloeosporioides s*.s.	SPL912	6/20	20/20	30	100
*C*. *cigarro*	SPL93	3/20	20/20	15	100
*C*. *ovataense*	T72	0	18/20	0	90
*C*. *siamense*	SPL2136	0	18/20	0	90
*C*. *sojae*	M72	0	16/20	0	80
*C*. *plurivorum*	SPML22	0	17/20	0	85
Control	H_2_O	0	0	0	0

**Fig 19:**
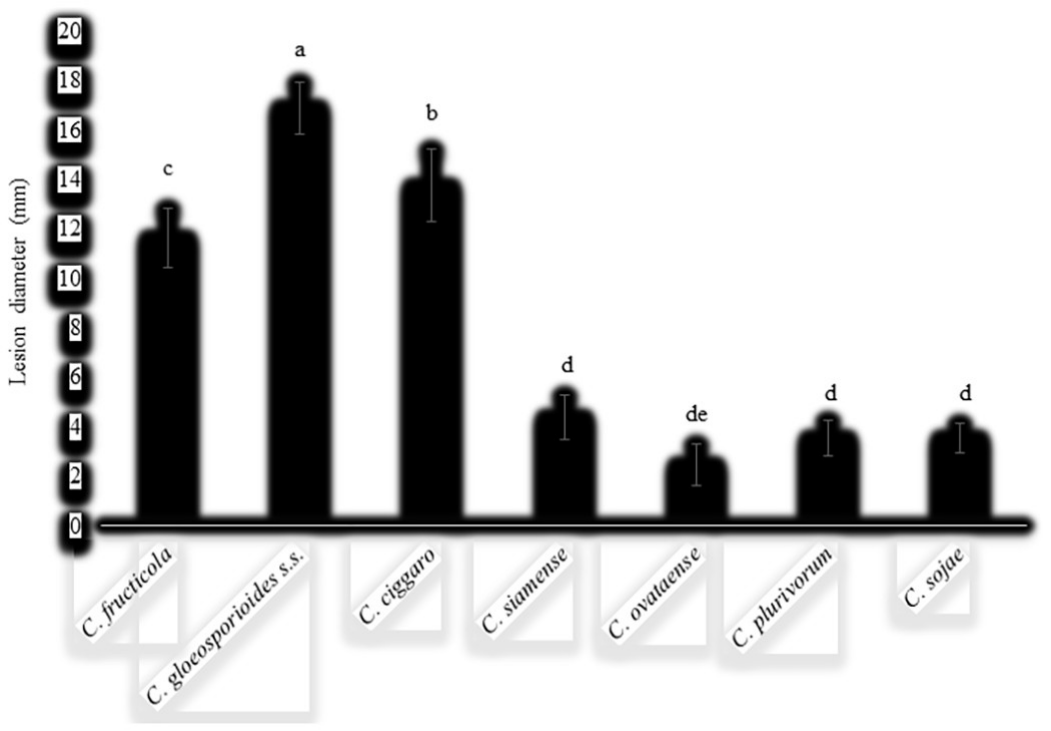
Lesion diameter on OLA leaves after wound-inoculation with different *Colletotrichum* species. Data were analyzed with SAS 9.4 by one-way ANOVA and means were compare using lsd test at the level of *P* = 0.05. Data (mean ± standard error) with different letters are significantly different at *P* < 0.05 (n = 12).

The lesions produced under wounded conditions appeared at 2–3 dpi and were of different size after seven dpi (Fig 18). *C*. *plurivorum* SPML22 showed the lowest infection rate (16/20; 80%), while *C*. *fructicola* 3C2, *C*. *gloeosporioides s*.s. SPL912, and *C*. *cigarro* SPL93 presented the highest infection rate (20/20; 100%) (Table 3). Species such as *C*. *ovataense* T72, *C*. *plurivorum* SPML22, *C*. *siamense* SPL2136, and *C*. *sojae* M72 induced a small necrotic lesion, while this was bigger in *C*. *fructicola* 3C2, *C*. *gloeosporioides s*.s. SPL912, and *C*. *cigarro* SPL93 (Fig 18). All species initially formed a small black lesion, which quickly increased in size in *C*. *fructicola* 3C2, *C*. *gloeosporioides s*.s. SPL912, and *C*. *cigarro* SPL93. The differences between lesion sizes produced by different *Colletotrichum* spp. were significant (Fig 19).

For instance, *C*. *gloeosporioides s*.s. SPL912 produced the largest necrotic lesions (16.9 ± 1.2 mm in diameter), whereas *C*. *ovataense* T72 produced the smallest (2.5 ± 0.8 mm in diameter). No lesions were induced on the control sides of the leaves inoculated with sterile water.

Fungi were isolated from the induced lesions neighboring the asymptomatic regions from diseased leaf tissue. The grown colonies matched their original isolates in their morphology and ITS sequences.

## Discussion

The genus *Colletotrichum* was considered to be one of the top 10 plant pathogen based on its wide host range, ability to cause anthracnose in many economically important crops, and significance as a post-harvest pathogen [43, 44]. Previous study on *Colletotrichum gloeosporioides* associated with OLA anthracnose were not reliable, since the identification process was only based on morphological characteristics [4]. The overall study of *Colletotrichum* spp. is remarkably difficult because of its complexity and diversity. Morphological characteristics alone cannot catalogue *Colletotrichum* spp. [20, 44]. This study applied advanced methods including morphological and multi-locus phylogenetic analyses to identify species associated with OLA anthracnose in South Korea. Pathogenicity test revealed that all identified *Colletotrichum* species were pathogenic to OLA. We have confidently identified seven species belonging to three different *Colletotrichum* species complex including gloeosporioides (*C*. *fructicola*, *C*. *gloeosporioides*, *C*. *siamense*, and *C*. *cigarro*), orchidearum (*C*. *sojae* and *C*. *plurivorum*) and magnum (*C*. *ovataense* sp. nov.). Of these, only *C*. *gloeosporioides* was reported to be a causal agent of OLA anthracnose, while *C*. *fructicola*, *C*. *siamense*, *C*. *cigarro*, *C*. *sojae*, and *C*. *plurivorum* were reported to be associated with this disease for the first time. Most importantly, this study described *C*. *ovataense* as a new species in *C*. *magnum* species complex causing OLA anthracnose.

The majority of identified *Colletotrichum* species exhibited distinct morphological characteristics including their colony colors, shapes, sizes of conidia, ascospores, and appressoria (Figs 8–14). These morphological characteristics along with molecular data have been widely used to delimit *Colletotrichum* species [17–20, 34]. However, not all the identified species were able to form a sexual morph in culture, only *C*. *fructicola* and *C*. *plurivorum* produced ascospores on PDA. The size of conidia of most of the isolated species from OLA were shown to vary significantly (Fig 15). For instance, *C*. *ovataense* had the largest conidia amongst all species, while *C*. *siamense* produced the smallest conidia. It is important to notice that although the conidia of species in the *C*. *orchidearum* species complex are similar to those in *C*. *gloeosporioides* species complex [18, 34], they belong to different species complex, which had been shown by the multigene sequence analysis.

The morphology, especially conidia size, of the *Colletotrichum* species isolated from OLA showed differences compared to those of other plants. For example, the conidia of *C*. *gloeosporioides* isolates from OLA (SPL912; 14.8–20.5 μm) are longer than those from citrus (11.3–14.7 μm) [45]; and the isolate of *C*. *plurivorum* (SPLM22; 17.1–24.4 μm) have longer conidia than that from coffee (15–17 μm), lima bean (11.5–14 μm), or common bean (0.5–11.5 μm) [18]. This can be explained by the variation of morphological characteristics in response to the host species, growth media, and environmental condition [19, 34].

The pathogenicity of all the identified *Colletotrichum* species (except *C*. *ovataense*) associated with anthracnose of other hosts was confirmed by previous studies [17–20, 34, 46] In this study, *Colletotrichum* species/isolates were revealed to be pathogenic to OLA, but showed a different level of aggressiveness. *Colletotrichum fructicola*, *C*. *gloeosporioides*, and *C*. *cigarro* caused specific symptoms on leaves under both unwounded and wounded conditions, while other species including *C*. *ovataense*, *C*. *plurivorum*, *C*. *siamense*, *C*. *siamense*, and *C*. *sojae* only caused symptoms on wounded leaves. The quiescent infection is an important feature in *Colletotrichum* spp. [17]. The wounds in leaves break the quiescent infection and enhance the infectivity of *Colletotrichum* species [17, 19]. The spot caused by *C*. *fructicola*, *C*. *gloeosporioides*, and *C*. *cigarro* on unwounded leaves were smaller than those on wounded leaves. The species *C*. *gloeosporioides* caused the biggest spot on leaves when compared to other species. Notably, only isolates of *C*. *fructicola*, *C*. *gloeosporioides*, and *C*. *cigarro* showed 100% infectivity. Different aggressiveness of *Colletotrichum* spp. has been also reported in several studies [15–17, 19].

The *C*. *gloeosporioides* species complex consists of many closely-related species and most are plant pathogenic to a wide range of crops [34, 47]. Interestingly, different species can infect the same host [15–17, 19, 20]. In this study, we identified four species (*C*. *fructicola*, *C*. *gloeosporioides*, *C*. *cigarro*, and *C*. *siamense*) from OLA. Multigene sequences including GAPDH, TUB2, ApMat gene can be used for differentiation of species within this complex [34, 44]. Sequences of ITS, *GAPDH*, *ACT*, *TUB2*, *CHS-1*, and *ApMat* were used to delineate fungal isolates of *C*. *gloeosporioides s*.*l*.

Damm et al. [18] described in detail nine closely-related species within the *C*. *magnum* species complex. Only *C*. *brevisporum* and an undescribed species from two independent publications have more than one host, while other species are known to infect a single host species each [18]. The phylogenetic analysis using a concatenated sequence of *ITS*, *GAPDH*, *ACT*, and *TUB2* showed that the new species, *C*. *ovataense*, clustered with *C*. *magnum* species complex and, from a sister clade with *C*. *magnum* and *C*. *liaoningense*. The non-significant recombination level between *C*. *ovataense*, *C*. *magnum*, and *C*. *liaoningense* was confirmed by PHI test. The conidial size of *C*. *ovataense* was bigger than those of *C*. *magnum*, and *C*. *liaoningense* species (S3 Table). The *C*. *ovataense* isolates can be distinguished from *C*. *magnum* via *GAPDH* (24 bp). Pathogenicity tests revealed that *C*. *ovataense* is pathogenic to OLA leaves. The effect of this pathogen on plant growth and host range should be further analyzed.

Only eight closely-related species were so for described in the *C*. *orchidearum* complex [18]. Three species including *C*. *orchidearum*, *C*. *plurivorum*, *and C*. *sojae* present a wide host range, while the other five species are so far only known from one host to be host-specific [18]. Importantly, most of the species within this complex produce both sexual and asexual morphs in culture [18] and this study observed sexual and asexual morph of *C*. *plurivorum* in culture. According to Damm et al. [18], the concatenated sequences of ITS, *GAPDH*, *ACT*, *TUB2*, and *CHS-1* were used to delineate species within the *C*. *orchidearum* species complex.

## Conclusions

In conclusion, this study is the first systematic investigation of morphological, molecular, and biological characterization *Colletotrichum* spp. associated with OLA anthracnose in South Korea. *Colletotrichum fructicola*, *C*. *siamense*, *C*. *cigarro*, *C*. *plurivorum*, and *C*. *sojae* together with a novel species (*C*. *ovataense*) were reported for the first time as the causal agents of this disease. The outcome of this study may provide an important basis to develop sustainable management strategies of this disease.

## Supporting information

S1 Fig A maximum likelihood tree based on AptMat sequence alignment. Present isolates are indicated by the bold face. *Colletotrichum theobromicola* CBS 124945 is used as the outgroup.
(PPTX)

S2 Fig A Bayesian inference phylogenetic tree (50% majority consensus) of *Colletotrichum kahawae* and *C*. *ciggaro* based on ITS and AptMat sequences. The tree is rooted with C. aotearoa (ICMP 18537). The reference isolates were used from the study of Cabral et al. (2020).
(PPTX)

S1 Table GenBank accession numbers of *Colletotrichum gloeosporioides* complex used in this study for molecular data analyses. (XLSX)

S2 Table GenBank accession numbers of *Colletotrichum magnum* and *C*. *orchidearum* complex used in this study for molecular data analyses. (XLSX)

S3 Table Comparison of morphological characteristics of close species from the *Colletotrichum* magnum complex. (DOCX)

S1 Data Size of conidia. (XLSX)

S2 Data Mycelial growth rate of *Colletotrichum* spp. in different temperature. (XLSX)

S3 Data Mycelial growth rate of *Colletotrichum* spp. in different culture media. (XLSX)

S4 Data Lesion size obtained from pathogenicity test. (XLSX)
