# Supplementary material for: An account of Colletotrichum species associated with anthracnose of Atractylodes ovata in South Korea based on morphology and molecular data
Source: PLoS One. 2022 Jan 25;17(1):e0263084. doi: 10.1371/journal.pone.0263084 (PMC8789177; doi:10.1371/journal.pone.0263084)
Supplement: S1 Fig — Present isolates are indicated by the bold face. Colletotrichum theobromicola CBS 124945 is used as the outgroup. (PPTX) [file pone.0263084.s001.pptx]

## Slide 1
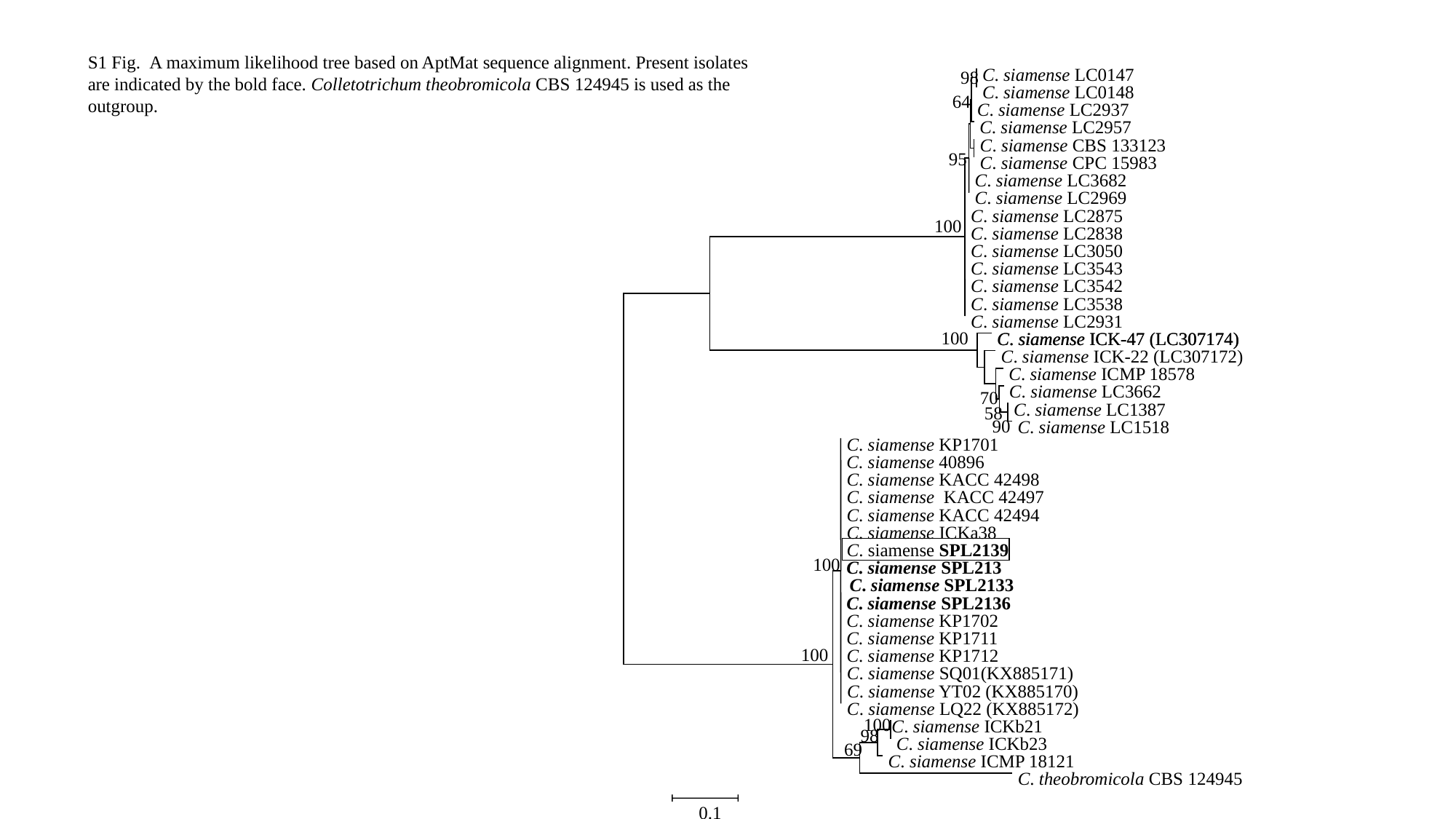

S1 Fig.  A maximum likelihood tree based on AptMat sequence alignment. Present isolates are indicated by the bold face. Colletotrichum theobromicola CBS 124945 is used as the outgroup.
 C. siamense LC0147
98
 C. siamense LC0148
64
 C. siamense LC2937
 C. siamense LC2957
 C. siamense CBS 133123
95
 C. siamense CPC 15983
 C. siamense LC3682
 C. siamense LC2969
 C. siamense LC2875
100
 C. siamense LC2838
 C. siamense LC3050
 C. siamense LC3543
 C. siamense LC3542
 C. siamense LC3538
 C. siamense LC2931
100
 C. siamense ICK-47 (LC307174)
 C. siamense ICK-47 (LC307174)
 C. siamense ICK-22 (LC307172)
 C. siamense ICMP 18578
 C. siamense LC3662
70
 C. siamense LC1387
58
90
 C. siamense LC1518
 C. siamense KP1701
 C. siamense 40896
 C. siamense KACC 42498
 C. siamense KACC 42497
 C. siamense KACC 42494
 C. siamense ICKa38
 C. siamense SPL2139
100
 C. siamense SPL213
 C. siamense SPL2133
 C. siamense SPL2136
 C. siamense KP1702
 C. siamense KP1711
100
 C. siamense KP1712
 C. siamense SQ01(KX885171)
 C. siamense YT02 (KX885170)
 C. siamense LQ22 (KX885172)
100
C. siamense ICKb21
98
 C. siamense ICKb23
69
 C. siamense ICMP 18121
 C. theobromicola CBS 124945
0.1
