# Supplementary material for: An account of Colletotrichum species associated with anthracnose of Atractylodes ovata in South Korea based on morphology and molecular data
Source: PLoS One. 2022 Jan 25;17(1):e0263084. doi: 10.1371/journal.pone.0263084 (PMC8789177; doi:10.1371/journal.pone.0263084)
Supplement: S2 Fig — The tree is rooted with C. aotearoa (ICMP 18537). The reference isolates were used from the study of Cabral et al. (2020). (PPTX) [file pone.0263084.s002.pptx]

## Slide 1
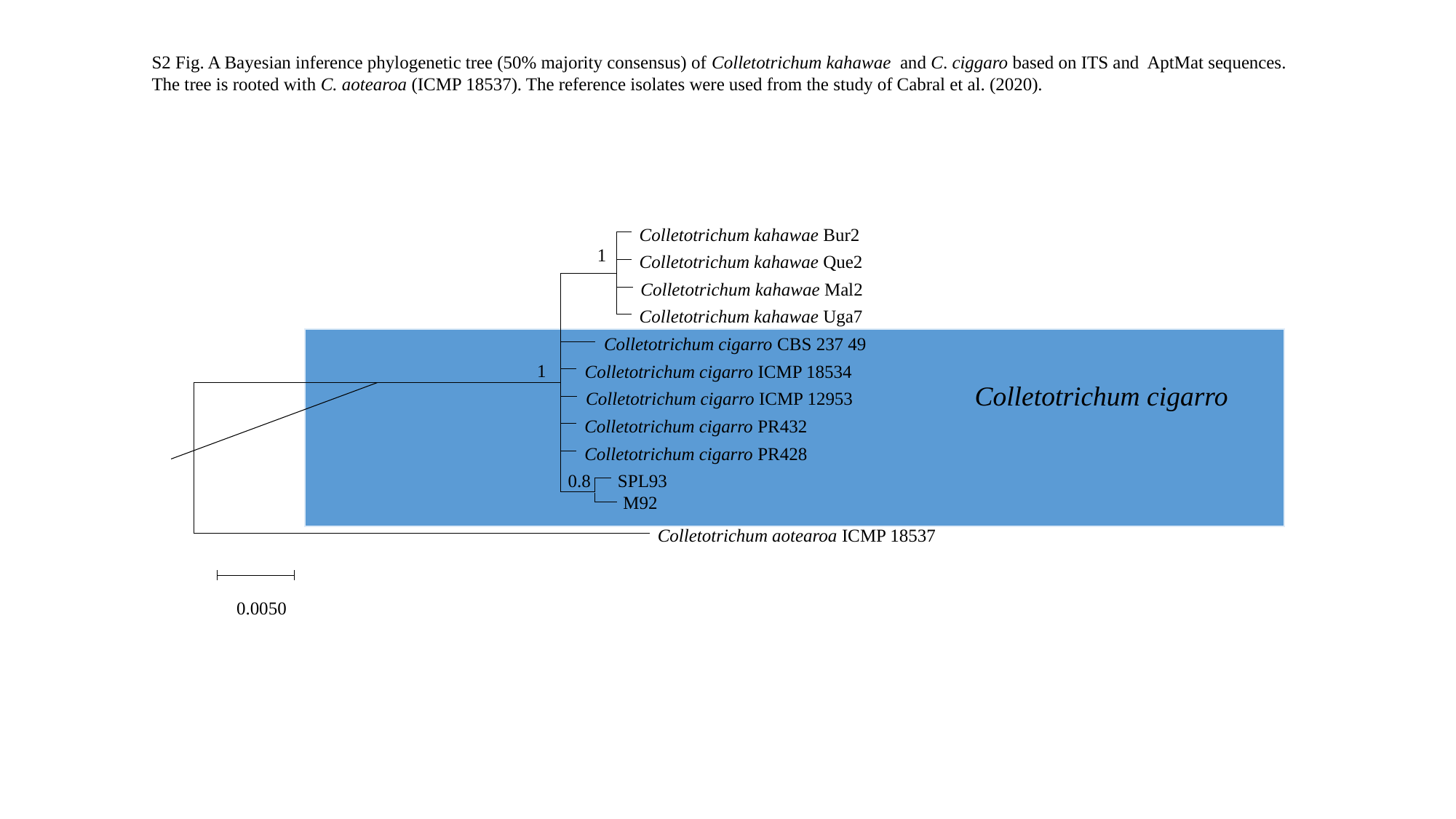

S2 Fig. A Bayesian inference phylogenetic tree (50% majority consensus) of Colletotrichum kahawae and C. ciggaro based on ITS and AptMat sequences. The tree is rooted with C. aotearoa (ICMP 18537). The reference isolates were used from the study of Cabral et al. (2020).
 Colletotrichum kahawae Bur2
 Colletotrichum kahawae Que2
 Colletotrichum kahawae Mal2
 Colletotrichum kahawae Uga7
 Colletotrichum cigarro CBS 237 49
 Colletotrichum cigarro ICMP 18534
 Colletotrichum cigarro ICMP 12953
 Colletotrichum cigarro PR432
 Colletotrichum cigarro PR428
 SPL93
 M92
 Colletotrichum aotearoa ICMP 18537
0.0050
1
1
Colletotrichum cigarro
0.8
