# Supplementary material for: An account of Colletotrichum species associated with anthracnose of Atractylodes ovata in South Korea based on morphology and molecular data
Source: PLoS One. 2022 Jan 25;17(1):e0263084. doi: 10.1371/journal.pone.0263084 (PMC8789177; doi:10.1371/journal.pone.0263084)
Supplement: S3 Table — (DOCX) [file pone.0263084.s005.docx]

| Colletotrichum  species | Strains | Teleomorph | | Anamorph | | References |
| --- | --- | --- | --- | --- | --- | --- |
|  |  | Asci | Ascospores | Appressoria | Conidia |  |
| *C*. *magnum* | CBS  519.97 | unitunicate, probably 8-spored,  cylindrical,  tapering to apex and base, smooth-walled, 71–122 ×  12–15.5 μm. | hyaline and aseptate but can  become pale brown and septate with age, smooth-walled,  allantoid, curved most in the middle, with rounded ends,  27–37 × 5–6.5 μm, mean ± SD =  32.1 ± 5.0 × 5.6 ± 0.7 μm, L/W ratio = 5.7. | single or in loose groups, medium to dark brown,  smooth-walled, ± circular, elliptical, clavate, spathulate or irregular  in outline, with an entire or undulate margin,  6.5–12.5 × 4.5–7.5 μm, mean ± SD = 9.4 ± 2.9 × 5.9 ± 1.5 μm | hyaline, smooth-walled, aseptate, straight, sometimes very slightly curved, cylindrical, the  apex and base rounded, 15.5–19 × 4–4.5 μm, mean ± SD = 17.4 ± 1.8 × 4.2 ± 0.2 μm, L/W ratio = 4.1 | Damm et al. 2019 |
| *C.* *ovataense* sp. nov. | KACC 49789 (= T72) | not observed | not observed | mostly single, pale to medium brown, smooth-walled, subglobose to globose, the edge entire, 8.5–14 × 7.5–12 μm (mean ± SD = 11.7 ± 1.5 × 8.8 ± 1.1), L/W ratio = 1.3. | hyaline, smooth-walled, aseptate, straight, cylindrical with both ends round and one end round and one end acute, 18.5 –26 × 4.5 – 7.0 μm (mean ± SD = 22.5 ± 1.8 × 5.5 ± 0.7 μm), L/W ratio = 3.7 | Present study |
| *C*. *liaoningense* | CGMCC3.17616 = CAUOS2 = LC6228 | not observed | not observed | single, medium to dark brown, aseptate, mostly ellipsoidal to irregular in outline, and crenate at margin, 3.5–5 × 2.5–4.5 μm ( mean = 4.1 × 2.9 μm), L/W ratio = 1.4. | cylindrical to clavate, both ends rounded or one end acute, contents granular and mostly present at the polar ends, hyaline, aseptate, smooth-walled, 14–18.5 μm × 5–7.5 μm ( mean = 16.3 × 6.1 μm), L/W ratio = 2.7. | Diao et al. 2017 |

S3Table. Comparison of morphological characteristics of close species from the *Colletotrichum magnum* complex
